# The burden of infective encephalitis in children in Asian countries (1990–2021): systematic analysis and projection of the burden of disease

**DOI:** 10.3389/fcimb.2025.1682224

**Published:** 2025-11-20

**Authors:** Yonghan Luo, Yuemei Feng, Yanchun Wang, Xiaotao Yang, Yan Guo, Yue Feng, Xueshan Xia

**Affiliations:** 1 Faculty of Life Science and Technology, Kunming University of Science and Technology, Kunming, Yunnan, China; 2 Second Department of Infectious Diseases, Kunming Children’s Hospital, Kunming, Yunnan, China; 3 Department of Nutrition and Food Hygiene, School of Public Health, Kunming Medical University, Kunming, China; 4 Department of Reproductive Gynecology, NHC Key Laboratory of Healthy Birth and Birth Defect Prevention in Western China, First People’s Hospital of Yunnan Province, Kunming, Yunnan, China; 5 Yunnan Provincial Key Laboratory of Public Health and Biosafety, Kunming Medical University, Kunming, Yunnan, China

**Keywords:** infective encephalitis, pediatrics, Asia, disease burden, Global Burden of Disease (GBD)

## Abstract

**Background:**

Infective encephalitis (IE) constitutes a severe neurological disorder with considerable morbidity and mortality among children, particularly in Asia, where stark disparities in socioeconomic status and healthcare accessibility prevail. Despite its substantial impact, comprehensive epidemiological investigations into pediatric IE across Asia remain scarce.

**Objective:**

This study aimed to systematically evaluate the burden of pediatric IE in Asia from 1990 to 2021, examine its association with sociodemographic development, identify major risk factors, and forecast future trends.

**Methods:**

Utilizing data from the Global Burden of Disease (GBD) 2021 study, we retrieved incidence, mortality, and disability-adjusted life years (DALYs) for individuals aged 0–14 years across six Asian regions and more than 50 countries. Statistical methods included trend analysis via estimated annual percentage change (EAPC), Spearman’s correlation between disease burden and the Sociodemographic Index (SDI), and decomposition of risk factors. A Bayesian Age-Period-Cohort (BAPC) model was employed to project trends in China, Japan, South Korea, and India from 2022 to 2035.

**Results:**

In 2021, Asia recorded 537,750 IE cases (49.3 per 100,000), 18,844 deaths (1.7 per 100,000), and 1.69 million DALYs (155.1 per 100,000). Between 1990 and 2021, there were substantial reductions in the burden of IE: incidence declined by 28.1% (from 68.6 to 49.3 per 100,000), mortality by 53.2% (from 3.7 to 1.7 per 100,000), and DALYs by 53.2% (from 331.0 to 155.1 per 100,000). South Asia exhibited the highest burden (71.7 incidence, 2.5 mortality, 226.7 DALYs per 100,000), whereas the High-income Asia Pacific region reported the lowest (11.7, 0.1, 11.6 per 100,000). SDI demonstrated strong inverse correlations with incidence (r = −0.73), mortality (r = −0.87), and DALYs (r = −0.87). Low birth weight/prematurity and particulate matter pollution emerged as principal risk factors. Forecasts suggest continued declines by 2035, with India and China projected to see the most pronounced reductions in incidence (−62.4% and −41.3%, respectively).

**Conclusion:**

Although the burden of IE has markedly declined since 1990, it remains a pressing public health concern in low-SDI Asian regions. Focused strategies addressing undernutrition, environmental hazards, and neonatal health are imperative.

## 1 Introduction

Central nervous system (CNS) infections represent one of the most severe categories of infectious diseases in children, with meningitis and encephalitis being the most prevalent forms (Kim, 2010; Giovane and Lavender, 2018; Messacar et al., 2018). Meningitis primarily involves inflammation of the meninges or spinal cord membranes, whereas encephalitis affects the brain parenchyma and is typically associated with more severe neurological damage, higher mortality rates, and poorer prognoses (Richie and Josephson, 2015; Navarro-Flores et al., 2022). Due to the immaturity of the blood-brain barrier and the underdeveloped immune system in children, their susceptibility to CNS infections is significantly higher compared to adults (Pang et al., 2025). Furthermore, children’s limited ability to articulate symptoms often leads to atypical clinical presentations, resulting in potential delays in diagnosis and treatment, thereby increasing the risk of irreversible neurological sequelae (Zainel et al., 2021; du Preez et al., 2025). Among CNS infections, encephalitis generally exhibits higher mortality and disability rates than meningitis, making it a major challenge in pediatric neuroinfectious diseases (Chang et al., 2007).

CNS infections contribute substantially to morbidity and mortality worldwide, particularly in developing countries. Asia, as the most densely populated continent, exhibits considerable regional variation in the burden of CNS infections (Global, regional, and national burden of meningitis and its aetiologies, 1990-2019: a systematic analysis for the Global Burden of Disease Study 2019, 2023). Regions such as South Asia, Central Asia, and Southeast Asia, characterized by lagging socioeconomic development and limited healthcare resources, bear a heavier disease burden. In contrast, developed countries like Japan, South Korea, and Singapore report lower incidence rates of infective encephalitis (IE), attributable to better sanitary conditions, widespread vaccination programs, and advanced medical technologies. However, existing epidemiological studies (Feigin et al., 2021; Johnson et al., 2023; Halsby et al., 2024; Ray et al., 2025) on IE have predominantly focused on Africa, the Americas, and Europe, with limited research addressing the epidemiological characteristics of pediatric IE in Asia. Moreover, the current studies on childhood IE in Asia have primarily concentrated on etiology, pathogens, and clinical outcomes (Chow and Dehority, 2021; Rocha et al., 2023), with a paucity of systematic investigations into regional trends of CNS infections across the continent. Although research (Global, regional, and national burden of meningitis and its aetiologies, 1990-2019: a systematic analysis for the Global Burden of Disease Study 2019, 2023) on meningitis among children is relatively abundant, studies on IE remain scarce, leading to an underestimation of the disease burden in this high-risk population.

The Global Burden of Disease (GBD) study (Murray, 2022; Global incidence, prevalence, years lived with disability (YLDs), disability-adjusted life-years (DALYs), and healthy life expectancy (HALE) for 371 diseases and injuries in 204 countries and territories and 811 subnational locations, 1990-2021: a systematic analysis for the Global Burden of Disease Study 2021, 2024) is one of the most comprehensive health databases available, encompassing incidence, mortality, and disability-adjusted life years (DALYs) data from 204 countries and regions, stratified by age, sex, and geographic location. It provides a standardized and validated approach for quantifying disease trends and attributable risks across populations. Previous GBD-based investigations have demonstrated the robustness of this methodology in capturing longitudinal patterns and cross-regional differences (Li et al., 2025). Utilizing GBD data, this study systematically assessed the disease burden of IE among children aged 0–14 years in Asia from 1990 to 2021, analyzing trends in incidence, mortality, and DALYs, and exploring the relationship between the Sociodemographic Index (SDI) and disease burden, as well as the impact of associated risk factors. Additionally, four socioeconomically representative countries—China, Japan, South Korea, and India—were selected, and the Bayesian Age-Period-Cohort (BAPC) model was employed to predict the disease burden in these countries up to 2035, aiming to provide an evidence-based foundation for the precise prevention and control of pediatric encephalitis in Asia.

## 2 Methodology

### 2.1 Data sources

This study utilized the GBD 2021 dataset, incorporating data on IE among children aged 0–14 years in Asia from 1990 to 2021. Key indicators of the disease burden, including incidence rates, mortality rates, and DALYs, were extracted. As this research involved a secondary analysis of publicly available data (https://ghdx.healthdata.org/gbd-2021). Ethics Review Committee of the Children’s Hospital of Kunming Medical University determined that the study did not need approval because it used publicly available data.

### 2.2 Retrieval method

The GBD Results Tool was employed for data extraction, with the search parameters configured as follows: the analytical measure selected were “Incidence”, “Death”, and “DALY”, with metric standards set as “Number” and “Rate”. The location scope was restricted to six Asian regions—Central Asia, High-income Asia Pacific, South Asia, East Asia, Southeast Asia, and North Africa and the Middle East—as well as over 50 specific Asian countries. The disease category selected was “Encephalitis” under “other infectious diseases”. Age groups included “0–12 months”,”12–24 months”,”2–4 years”,”5–9 years”, and “10–14 years”. The study period was set from “1990 to 2021”. Risk factors encompassed all detailed factors within the GBD database, including environmental, metabolic, and behavioral risks.

### 2.3 Key definitions

The SDI is a composite indicator developed by the GBD to assess the level of economic and social development of a country or region (Global burden of 369 diseases and injuries in 204 countries and territories, 1990-2019: a systematic analysis for the Global Burden of Disease Study 2019, 2020; Global incidence, prevalence, years lived with disability (YLDs), disability-adjusted life-years (DALYs), and healthy life expectancy (HALE) for 371 diseases and injuries in 204 countries and territories and 811 subnational locations, 1990-2021: a systematic analysis for the Global Burden of Disease Study 2021, 2024). It incorporates data on per capita income, average educational attainment, and fertility rates, assigning an SDI value ranging from 0 to 1, with higher values indicating a higher level of development.

### 2.4 Statistical analysis

Data analysis and visualization in this study were conducted using R statistical software, version 4.4.1. Incidence rates, mortality rates, DALYs, and their corresponding ratios served as the primary indicators for evaluating the burden of IE among children. Each rate was reported per 100,000 population according to the GBD methodology and was accompanied by a 95% uncertainty interval (UI). The estimated annual percentage change (EAPC) was calculated to assess trends in the disease burden, with positive values indicating an increasing trend and negative values indicating a decreasing trend.

Spearman’s rank correlation test was employed to evaluate the linear relationship between the SDI and incidence, mortality, and DALY rates across different Asian regions and countries. To forecast the future trend of pediatric IE, the BAPC model was used to predict the incidence and mortality rates among children aged 0–14 years in China, Japan, South Korea, and India from 2022 to 2035, providing a prospective basis for the formulation of disease prevention and control strategies. A p-value of less than 0.05 was considered statistically significant.

## 3 Results

### 3.1 Incidence, mortality, and DALYs of infectious encephalitis among children in Asia

#### 3.1.1 Incidence

In 2021, the total number of incident cases of IE in Asia was 537,750.447 (95% UI: 458,677.367–633,964.538), corresponding to an incidence rate of 49.337 (95% UI: 42.082–58.164) per 100,000 population. Among males, the number of cases was 303,271.903 (95% UI: 259,234.200–356,621.823), while females accounted for 234,478.544 cases (95% UI: 198,874.130–275,918.669). The number of cases among males was approximately 1.29 times that of females, with the male incidence rate (53.286 per 100,000) slightly exceeding that of females (45.022 per 100,000) (see **Table 1**).

**Table 1:** Encephalitis burden of children of different ages and sexes in Asia from 1990 to 2021.

Index	Level	1990		2021		1990–2021	
		Number (95%UI)	Rate per 100,000(95%UI)	Number (95%UI)	Rate per 100,000(95%UI)	Rate change	Rate EAPC(95%CI)
Incidence	Asia	727863.117(627381.792-852659.015)	68.623(59.150-80.389)	537750.447(458677.367-633964.538)	49.337(42.082-58.164)	-0.281(-0.312–0.253)	-1.22(-1.33,-1.11)
	Central Asia	5949.748(5208.983-6836.226)	23.807(20.843-27.355)	5372.164(4673.062-6201.372)	19.411(16.885-22.407)	-0.185(-0.211–0.156)	-0.56(-0.66,-0.46)
	East Asia	160689.256(134409.376-193131.145)	48.718(40.751-58.554)	119319.031(97882.754-144973.083)	44.630(36.612-54.226)	-0.084(-0.156–0.019)	0.22(-0.06,0.49)
	High-income Asia Pacific	4749.952(3594.734-6186.142)	13.494(10.212-17.574)	2630.975(1982.404-3463.485)	11.732(8.840-15.444)	-0.131(-0.156–0.109)	0.02(-0.29,0.34)
	North Africa and Middle East	19393.967(15948.846-23773.613)	13.805(11.353-16.922)	21550.408(17433.449-26704.633)	11.755(9.510-14.567)	-0.148(-0.178–0.119)	-0.47(-0.51,-0.44)
	South Asia	502491.683(437585.002-582290.202)	115.952(100.975-134.366)	363320.568(313296.014-423058.832)	71.658(61.791-83.440)	-0.382(-0.409–0.352)	-1.95(-2.11,-1.79)
	Southeast Asia	45377.140(37594.544-55541.077)	26.576(22.018-32.528)	35950.051(30078.362-43196.155)	20.822(17.421-25.019)	-0.216(-0.244–0.187)	-0.97(-1.06,-0.88)
	Gender						
	Male	374412.166(322942.874-439954.509)	68.151(58.782-80.081)	303271.903(259234.200-356621.823)	53.286(45.548-62.660)	-0.218(-0.250–0.188)	-0.91(-1.01,-0.82)
	Female	353450.951(305256.057-413217.757)	69.130(59.704-80.820)	234478.544(198874.130-275918.669)	45.022(38.185-52.979)	-0.349(-0.379–0.321)	-1.58(-1.71,-1.46)
	Age						
	<1 year	131087.567(111366.575-152675.697)	172.104(146.212-200.447)	64823.388(55832.729-74563.655)	102.778(88.523-118.221)	-0.403(-0.421–0.384)	-2.08(-2.23,-1.93)
	12–23 months	92003.273(74434.506-112264.788)	122.923(99.450-149.994)	45426.405(37263.186-55298.400)	69.531(57.036-84.642)	-0.434(-0.456–0.410)	-2.20(-2.35,-2.04)
	2–4 years	206885.797(163360.435-255873.331)	92.343(72.916-114.209)	136315.596(104992.102-169117.297)	63.747(49.099-79.086)	-0.310(-0.349–0.274)	-1.42(-1.56,-1.29)
	5–9 years	192829.924(142507.570-253181.434)	53.774(39.741-70.604)	179315.208(138676.099-228291.728)	47.674(36.869-60.695)	-0.113(-0.148–0.068)	-0.56(-0.66,-0.46)
	10–14 years	105056.555(78245.928-143082.846)	32.125(23.927-43.754)	111869.850(83978.813-148746.771)	30.107(22.601-40.031)	-0.063(-0.102–0.013)	-0.47(-0.59,-0.35)
Deaths	Asia	39174.371(28191.084-47692.273)	3.693(2.658-4.496)	18844.092(13582.286-24558.221)	1.729(1.246-2.253)	-0.532(-0.681–0.293)	-2.43(-2.62,-2.24)
	Central Asia	677.822(573.984-814.710)	2.712(2.297-3.260)	458.846(363.891-577.640)	1.658(1.315-2.087)	-0.389(-0.547–0.196)	-1.45(-1.82,-1.09)
	East Asia	10303.342(6711.579-12995.564)	3.124(2.035-3.940)	1556.407(1158.714-2261.883)	0.582(0.433-0.846)	-0.814(-0.876–0.654)	-5.20(-5.55,-4.85)
	High-income Asia Pacific	64.815(58.064-84.957)	0.184(0.165-0.241)	27.881(24.351-31.690)	0.124(0.109-0.141)	-0.325(-0.531–0.203)	-2.02(-2.63,-1.42)
	North Africa and Middle East	1583.763(1190.024-2099.319)	1.127(0.847-1.494)	1088.442(844.926-1396.263)	0.594(0.461-0.762)	-0.473(-0.626–0.300)	-1.75(-1.86,-1.65)
	South Asia	23219.127(16269.426-29777.995)	5.358(3.754-6.871)	12827.458(8973.507-17609.483)	2.530(1.770-3.473)	-0.528(-0.692–0.203)	-2.59(-2.76,-2.42)
	Southeast Asia	4365.072(2715.074-6175.339)	2.556(1.590-3.617)	3451.072(1883.925-4536.928)	1.999(1.091-2.628)	-0.218(-0.517-0.089)	-0.69(-0.75,-0.62)
	Gender						
	Male	17237.325(8649.489-23595.515)	3.138(1.574-4.295)	9756.848(5626.957-13248.222)	1.714(0.989-2.328)	-0.454(-0.742–0.129)	-1.95(-2.07,-1.82)
	Female	21937.045(15053.521-28114.683)	4.291(2.944-5.499)	9087.244(7096.241-11991.003)	1.745(1.363-2.302)	-0.593(-0.711–0.261)	-2.86(-3.11,-2.61)
	Age						
	<1 year	13199.067(9274.267-16102.293)	17.329(12.176-21.141)	6374.389(4271.434-9055.464)	10.107(6.772-14.357)	-0.417(-0.617–0.069)	-2.15(-2.38,-1.92)
	12–23 months	7262.074(4857.111-9603.789)	9.703(6.489-12.831)	2782.987(1879.462-3761.766)	4.260(2.877-5.758)	-0.561(-0.721–0.251)	-3.14(-3.34,-2.95)
	2–4 years	10184.743(6193.545-13987.904)	4.546(2.764-6.243)	3536.818(2261.606-5011.283)	1.654(1.058-2.343)	-0.636(-0.787–0.329)	-3.59(-3.86,-3.31)
	5–9 years	5290.242(3825.968-6680.993)	1.475(1.067-1.863)	3230.916(2297.249-4127.738)	0.859(0.611-1.097)	-0.418(-0.580–0.231)	-1.49(-1.91,-1.07)
	10–14 years	3238.245(2434.297-3964.280)	0.990(0.744-1.212)	2918.982(2246.245-3706.208)	0.786(0.605-0.997)	-0.207(-0.398-0.065)	-0.67(-0.93,-0.41)
DALY	Asia	3511232.247(2567526.151-4245444.153)	331.040(242.067-400.261)	1690261.197(1223965.925-2174493.694)	155.077(112.295-199.503)	-0.532(-0.677–0.300)	-2.44(-2.62,-2.26)
	Central Asia	59605.701(50420.803-71652.200)	238.507(201.754-286.710)	40403.467(32151.375-50841.200)	145.988(116.171-183.703)	-0.388(-0.546–0.194)	-1.44(-1.80,-1.08)
	East Asia	929891.610(611831.930-1167148.405)	281.927(185.497-353.860)	148346.962(114209.673-210771.926)	55.487(42.719-78.837)	-0.803(-0.865–0.646)	-5.03(-5.36,-4.70)
	High-income Asia Pacific	5998.686(5397.322-7682.753)	17.042(15.334-21.826)	2599.496(2295.962-2927.531)	11.592(10.238-13.054)	-0.320(-0.514–0.206)	-1.92(-2.48,-1.36)
	North Africa and Middle East	139559.209(105149.780-184471.383)	99.341(74.847-131.310)	96431.312(75896.844-122960.242)	52.602(41.401-67.073)	-0.470(-0.618–0.302)	-1.74(-1.84,-1.64)
	South Asia	2080786.195(1477564.315-2651770.394)	480.150(340.954-611.907)	1149445.910(818484.269-1552712.978)	226.706(161.430-306.242)	-0.528(-0.687–0.214)	-2.61(-2.77,-2.44)
	Southeast Asia	386899.387(242903.009-547596.866)	226.591(142.259-320.706)	303022.149(166522.252-398289.373)	175.509(96.449-230.687)	-0.225(-0.519-0.078)	-0.72(-0.79,-0.66)
	Gender						
	Male	1547965.751(799277.717-2097536.720)	281.762(145.485-381.796)	879180.811(522700.304-1182280.712)	154.475(91.840-207.731)	-0.452(-0.732–0.147)	-1.95(-2.07,-1.84)
	Female	1963266.495(1358022.438-2502326.535)	383.990(265.612-489.423)	811080.386(637847.007-1062839.511)	155.734(122.472-204.074)	-0.594(-0.708–0.272)	-2.88(-3.13,-2.64)
	Age						
	<1 year	1184485.193(832926.050-1444329.335)	1555.103(1093.543-1896.250)	572014.148(383709.978-812167.652)	906.932(608.374-1287.696)	-0.417(-0.616–0.069)	-2.15(-2.38,-1.92)
	12–23 months	646747.323(433729.308-853105.149)	864.102(579.494-1139.812)	248031.548(167978.592-334430.545)	379.645(257.114-511.890)	-0.561(-0.720–0.252)	-3.14(-3.34,-2.95)
	2–4 years	900714.590(553161.547-1227883.411)	402.032(246.903-548.064)	314113.455(205313.157-441082.454)	146.892(96.013-206.268)	-0.635(-0.783–0.335)	-3.57(-3.84,-3.30)
	5–9 years	482322.801(358733.255-594970.653)	134.504(100.039-165.918)	294711.268(217428.087-369970.876)	78.353(57.807-98.362)	-0.417(-0.569–0.248)	-1.53(-1.91,-1.14)
	10–14 years	296962.340(232458.811-355598.857)	90.809(71.084-108.739)	261390.778(208746.854-325182.038)	70.346(56.178-87.514)	-0.225(-0.387-0.001)	-0.79(-1.02,-0.57)

**Figure 1:**
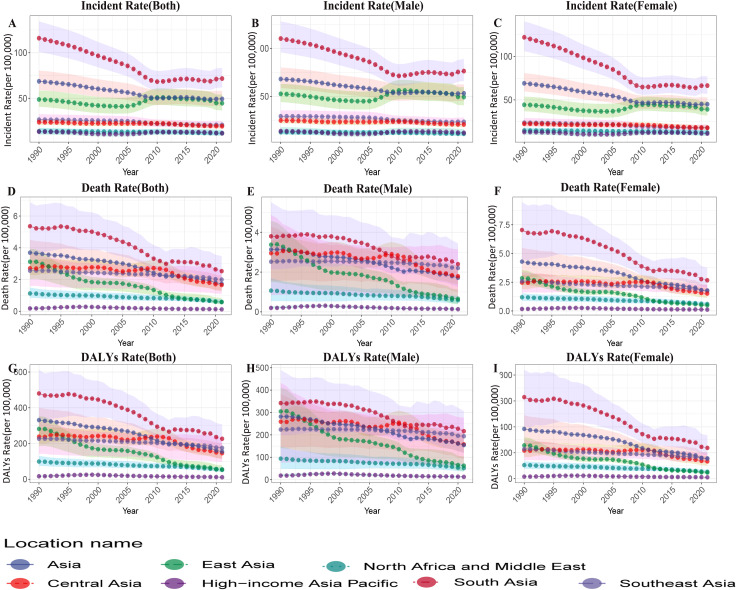
Epidemiological trends in the incidence, prevalence, and disability-adjusted life years (DALYs) of childhood encephalitis by sex across various Asian regions from 1990 to 2021. **(A)** Incident rate (Both), **(B)** Incident rate (Male), **(C)** Incident rate (Female), **(D)** Death rate (Both), **(E)** Death rate (Male), **(F)** Death rate (Female), **(G)** DALYs rate (Both), **(H)** DALYs rate (Male), **(I)** DALYs rate (Female).

From 1990 to 2021, the overall incidence rate in Asia showed a significant downward trend (see **Figures 1A–C**), decreasing from 68.623 (95% UI: 59.150–80.389) to 49.337 (95% UI: 42.082–58.164) per 100,000 population, with an EAPC of -1.22 (95% CI: -1.33 to -1.11). In terms of sex-specific trends, females exhibited a greater decline compared to males (female EAPC: -1.58; 95% CI: -1.71 to -1.46 vs. male EAPC: -0.91; 95% CI: -1.01 to -0.82).

**Figure 2:**
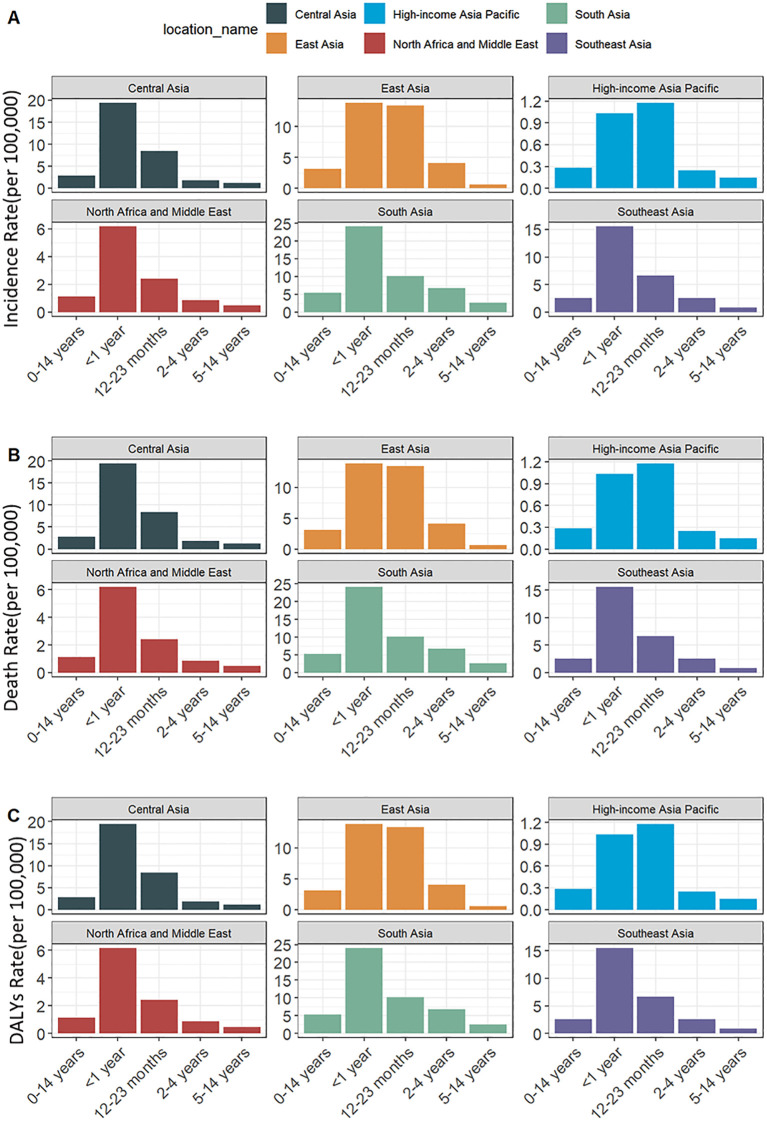
Incidence, mortality, and disability-adjusted life years (DALYs) associated with the burden of childhood encephalitis across different age groups in Asian countries in 2021. **(A)** Incidence rate (per 100,000), **(B)** Death rate (per 100,000), **(C)** DALYs rate (per 100,000).

Age-specific analysis revealed that the decline in incidence was most pronounced in younger age groups, particularly among children aged 12–23 months (EAPC: -2.20; 95% CI: -2.35 to -2.04) and infants under 1 year old (EAPC: -2.08; 95% CI: -2.23 to -1.93) (see **Figure 2A**).

#### 3.1.2 Mortality

In 2021, the total number of deaths due to IE in Asia was 18,844.092 (95% UI: 13,582.286–24,558.221), corresponding to a mortality rate of 1.729 (95% UI: 1.246–2.253) per 100,000 population. Among males, deaths totaled 9,756.848 (95% UI: 5,626.957–13,248.222) with a mortality rate of 1.714 (95% UI: 0.989–2.328) per 100,000, while females accounted for 9,087.244 deaths (95% UI: 7,096.241–11,991.003) with a mortality rate of 1.745 (95% UI: 1.363–2.302) per 100,000. Although the number of male deaths slightly exceeded that of females, the mortality rate among females was marginally higher, with a mortality rate ratio of 1.02 (1.745/1.714) (see **Table 1**).

Between 1990 and 2021, the mortality rate in Asia markedly decreased (see **Figures 1D–F**), from 3.693 (95% UI: 2.658–4.496) to 1.729 (95% UI: 1.246–2.253) per 100,000, with an EAPC of -2.43 (95% CI: -2.62 to -2.24). The decline was more substantial among females (EAPC: -2.86; 95% CI: -3.11 to -2.61) than males (EAPC: -1.95; 95% CI: -2.07 to -1.82). The most significant reductions were observed in the younger age groups, particularly among children aged 2–4 years (EAPC: -3.59; 95% CI: -3.86 to -3.31) and those aged 12–23 months (EAPC: -3.14; 95% CI: -3.34 to -2.95) (see **Figure 2B**).

#### 3.1.3 DALYs

Regarding DALYs, the total number of DALYs attributable to IE in Asia in 2021 was 1,690,261.197 (95% UI: 1,223,965.925–2,174,493.694), corresponding to a DALY rate of 155.077 (95% UI: 112.295–199.503) per 100,000 population. Among males, the total DALYs were 879,180.811 (95% UI: 522,700.304–1,182,280.712), with a DALY rate of 154.475 (95% UI: 91.840–207.731) per 100,000, while females accounted for 811,080.386 DALYs (95% UI: 637,847.007–1,062,839.511) with a DALY rate of 155.734 (95% UI: 122.472–204.074) per 100,000 (see **Table 1**).

The total DALYs were comparable between males and females, with females exhibiting a slightly higher DALY rate, reflected in a DALY rate ratio of 1.01 (155.734/154.475). During the study period, DALY rates in Asia showed a significant decline (see **Figures 1G–I**), from 331.040 (95% UI: 242.067–400.261) per 100,000 in 1990 to 155.077 (95% UI: 112.295–199.503) per 100,000 in 2021, corresponding to an EAPC of -2.44 (95% CI: -2.62 to -2.26). Like mortality trends, females experienced a more rapid decline (EAPC: -2.88; 95% CI: -3.13 to -2.64). The most pronounced reductions were observed among children aged 2–4 years (EAPC: -3.57; 95% CI: -3.84 to -3.30) and those aged 12–23 months (EAPC: -3.14; 95% CI: -3.34 to -2.95) (see **Figure 2C**).

### 3.2 Incidence, mortality, and DALYs of infectious encephalitis among children by Region in Asia

#### 3.2.1 Incidence

In 2021, South Asia reported the highest number of incident cases, reaching 363,320.568 (95% UI: 313,296.014–423,058.832), with an incidence rate of 71.658 (95% UI: 61.791–83.440) per 100,000 population. In contrast, the High-income Asia Pacific region reported the fewest cases, totaling 2,630.975 (95% UI: 1,982.404–3,463.485), with an incidence rate of 11.732 (95% UI: 8.840–15.444) per 100,000.

South Asia also showed the most rapid decline in incidence, with an EAPC of –1.95 (95% CI: –2.11 to –1.79). In comparison, the High-income Asia Pacific region had the slowest decline, with an EAPC of 0.02 (95% CI: –0.29 to 0.34) (see **Table 1**).

#### 3.2.2 Mortality

In 2021, South Asia again reported the highest number of deaths, totaling 12,827.458 (95% UI: 8,973.507–17,609.483), with a mortality rate of 2.530 (95% UI: 1.770–3.473) per 100,000 population. In contrast, the High-income Asia Pacific region had the fewest deaths, with 27.881 (95% UI: 24.351–31.690), corresponding to a mortality rate of 0.124 (95% UI: 0.109–0.141) per 100,000.

Among regions, East Asia exhibited the fastest decline in mortality rate, with an EAPC of –5.20 (95% CI: –5.55 to –4.85), while Southeast Asia showed the slowest decline with an EAPC of –0.69 (95% CI: –0.75 to –0.62) (see **Table 1**).

#### 3.2.3 DALYs

Regarding DALYs, South Asia recorded the highest total in 2021, reaching 1,149,445.910 (95% UI: 818,484.269–1,552,712.978), corresponding to a DALY rate of 226.706 (95% UI: 161.430–306.242) per 100,000 population. Conversely, the High-income Asia Pacific region reported the lowest DALY burden, with 2,599.496 (95% UI: 2,295.962–2,927.531) and a DALY rate of 11.592 (95% UI: 10.238–13.054) per 100,000.

In terms of trends, East Asia achieved the fastest decline in DALY rates, with an EAPC of –5.03 (95% CI: –5.36 to –4.70), while Southeast Asia experienced the slowest decline, with an EAPC of –0.72 (95% CI: –0.79 to –0.66) (see **Table 1**).

### 3.3 Incidence, mortality, and DALYs of pediatric infectious encephalitis in Asian countries

#### 3.3.1 Incidence

In 2021, India reported the highest number of pediatric IE cases, with approximately 272,432 children affected (95% UI: 236,255–316,191). However, the highest incidence rate was observed in Pakistan, with approximately 73.353 (95% UI: 62.328–86.004) cases per 100,000 population.

In contrast, Brunei Darussalam reported the fewest number of cases, with only about 9 infections (95% UI: 7–12), and Singapore had the lowest incidence rate, at 4.54 cases per 100,000 population.

**Figure 3:**
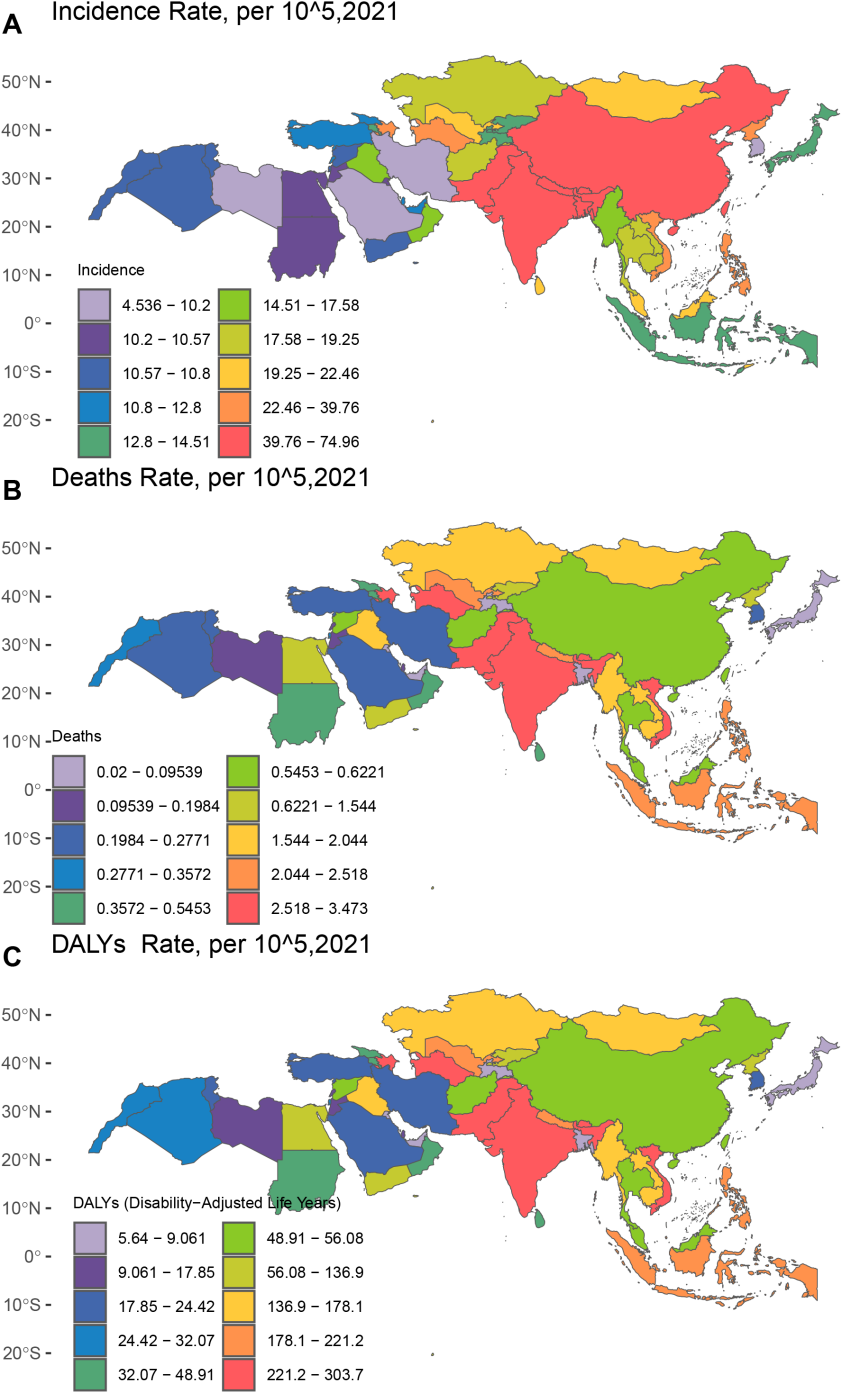
Incidence, mortality, and disability-adjusted life years (DALYs) associated with the burden of childhood encephalitis in Asian countries in 2021. **(A)** Incidence rate, per 10^5, **(B)** Death rate, per 10^5, **(C)** DALYs rate, per 10^5.

From 1990 to 2021, China experienced the slowest decline in incidence rate, with an EAPC of 0.23 (95% CI: –0.05 to 0.51). India showed the most significant decrease in incidence, with an EAPC of –2.36 (95% CI: –2.66 to –2.06) (see **Figure 3A**; **Supplementary Tables S1–S3**).

#### 3.3.2 Mortality

Between 1990 and 2021, mortality rates due to pediatric IE varied significantly across Asian countries. India reported the highest number of deaths in 2021, with 9,621 fatalities (95% UI: 6,653–13,633). Pakistan recorded the highest mortality rate, at 3.473 (95% UI: 1.753–5.276) per 100,000 population.

Bangladesh had the lowest mortality rate, at just 0.020 per 100,000 (95% UI: 0.007–0.091). In terms of trends, Palestine showed the slowest decline in mortality, with an EAPC of 3.54 (95% CI: 2.49–4.60), while Jordan demonstrated the most rapid reduction, with an EAPC of –5.88 (95% CI: –6.81 to –4.95) (see **Figure 3B**; **Supplementary Tables S4–S6**).

#### 3.3.3 DALYs

India consistently reported the highest number of DALYs attributable to pediatric IE, with a total of approximately 866,085 DALYs in 2021 (95% UI: 648,752.93–1,111,018.75), far exceeding any other country. Brunei had the lowest total DALYs, with only 5.67 (95% UI: 4.00–7.62).

In terms of DALY rates, Pakistan recorded the highest in 2021, at 303.703 (95% UI: 158.863–456.353) per 100,000 population. Conversely, Brunei Darussalam had the lowest DALY rate, at 5.665 (95% UI: 4.134–7.980) per 100,000.

Regarding long-term trends, Turkmenistan had the slowest decline in DALY rates since 1990, with an EAPC of +3.43 (95% CI: 2.32–4.54), whereas Jordan had the fastest reduction, with an EAPC of –5.59 (95% CI: –6.31 to –4.86) (see **Figure 3C** and **Supplementary Table S7–S9**).

### 3.4 Correlation between socio-demographic index and the burden of infectious encephalitis among children in Asia

In this study, Spearman’s rank correlation analysis was performed to evaluate the relationship between SDI and the burden of IE among children aged 0–14 years in Asia.

**Figure 4:**
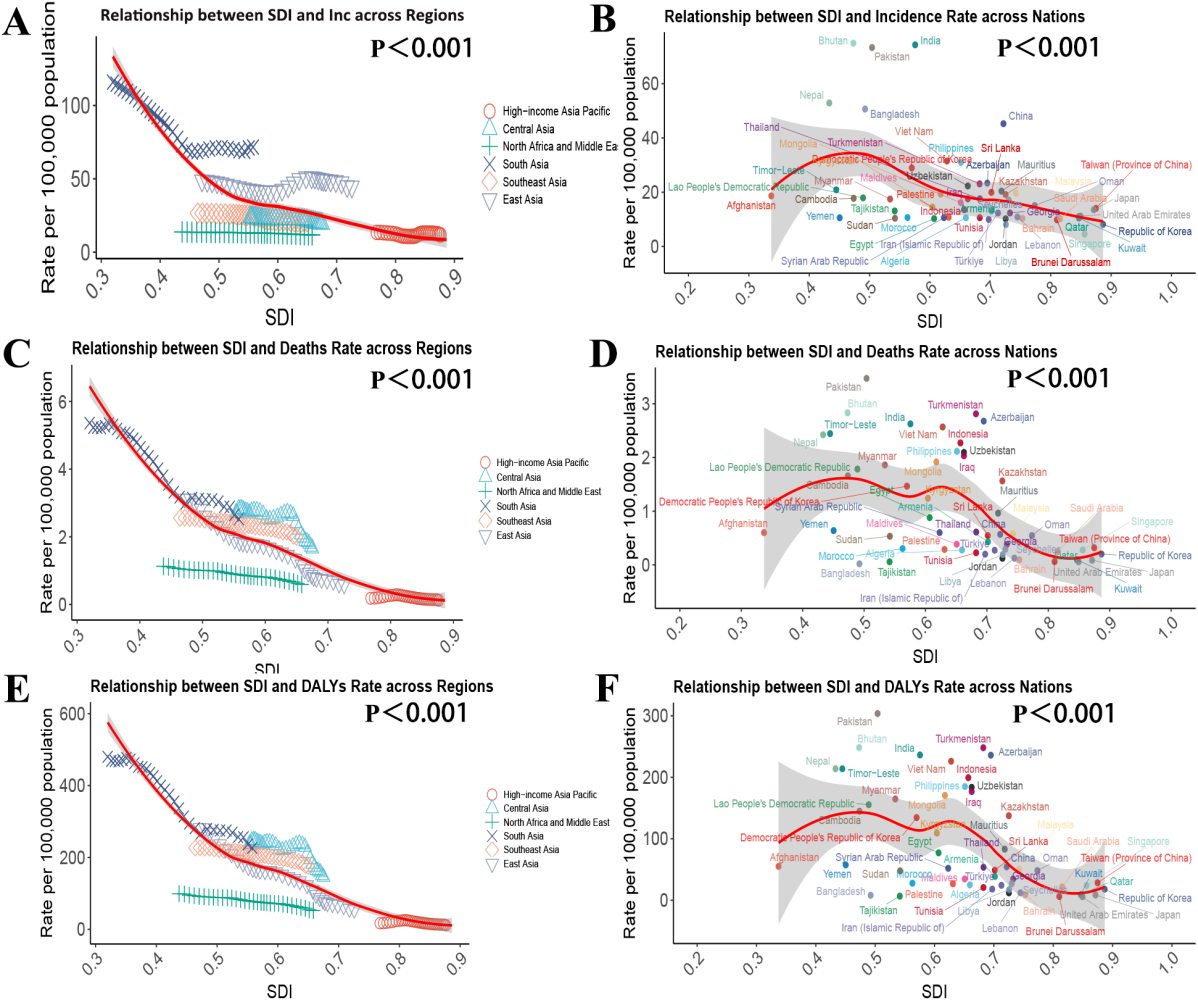
Relationship between the Socio-demographic Index (SDI) and the burden of encephalitis in Asian countries and regions in 2021. **(A)** Relationship between SDI and Incidence rate across regions, **(B)** Relationship between SDI and Incidence rate across nations, **(C)** Relationship between SDI and Death rate across regions, **(D)** Relationship between SDI and Death rate across nations, (E) Relationship between SDI and DALYs rate across regions, (F) Relationship between SDI and DALYs rate across nations.

The results demonstrated that SDI levels across Asian regions were significantly negatively correlated with incidence (r = –0.729, p < 0.001), mortality (r = –0.867, p < 0.001), and DALY rates (r = –0.868, p < 0.001) of IE among children (see **Figures 4A, C, E**).

Further analysis at the national level revealed that the SDI of individual countries was also significantly negatively correlated with incidence (r = –0.461, p < 0.001), mortality (r = –0.569, p < 0.001), and DALY rates (r = –0.584, p < 0.001) among children aged 0–14 years (see **Figures 4B, D, F**).

### 3.5 Risk factor analysis

The risk factor analysis identified low birth weight and preterm birth, as well as particulate matter pollution, as major risk factors contributing to mortality and DALYs associated with IE among children in Asian countries.

**Figure 5:**
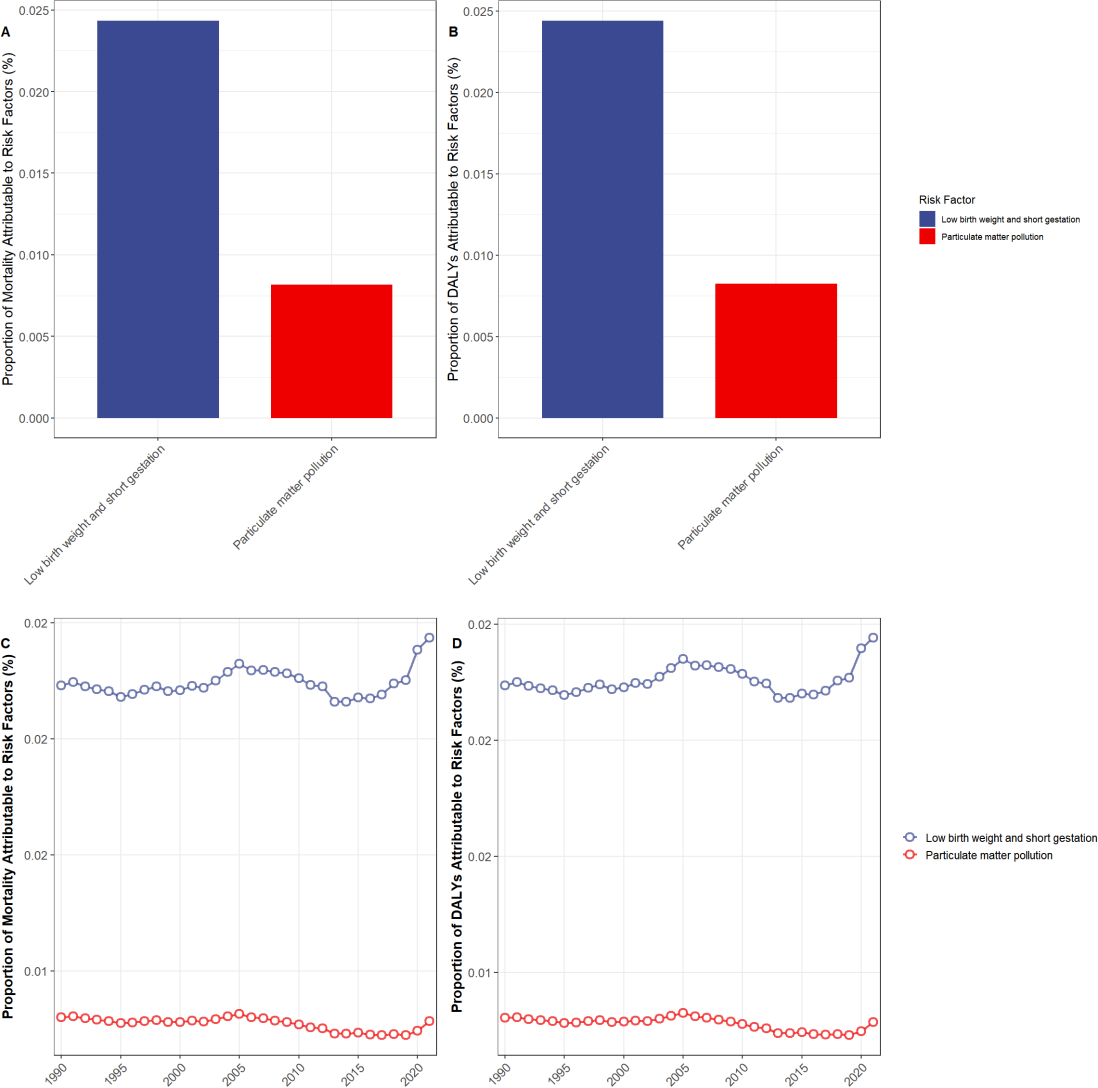
Risk factor analysis of encephalitis among children in Asia. **(A)** Risk factors associated with mortality in 2021; **(B)** Risk factors contributing to DALYs in 2021; **(C)** Temporal trends in mortality-related risk factors from 1990 to 2021; **(D)** Temporal trends in DALY-related risk factors from 1990 to 2021.

Low birth weight and preterm birth were the predominant risk factors, contributing a higher proportion of deaths compared to particulate matter pollution (0.024% vs. 0.007% in 2021, respectively) (see **Figures 5A, B**).

Between 1990 and 2019, the overall contribution of low birth weight and preterm birth and particulate matter pollution to the burden of pediatric encephalitis in Asia remained relatively stable (see **Figures 5C, D**).

### 3.6 Predictive analysis

Asia comprises 48 countries and 6 regions, with a total population of approximately 4.759 billion, accounting for 58% of the global population. Within this population, China (East Asia, ~1.4 billion) and India (South Asia, ~1.44 billion) together represent about 59% of Asia’s total population.

Given the significant differences in economic and social development across regions and countries, the burden of IE also varies accordingly. India and China, as the two most populous developing countries, rank first and second globally in terms of population size, respectively, while Japan and South Korea are representative developed countries in Asia.

Therefore, this study selected China, Japan, South Korea, and India as representative countries to predict trends in the incidence and mortality rates of pediatric IE from 2022 to 2035.

#### 3.6.1 Incidence prediction

The prediction results indicated a declining trend in the incidence of pediatric IE in China, Japan, South Korea, and India by 2035.

**Figure 6:**
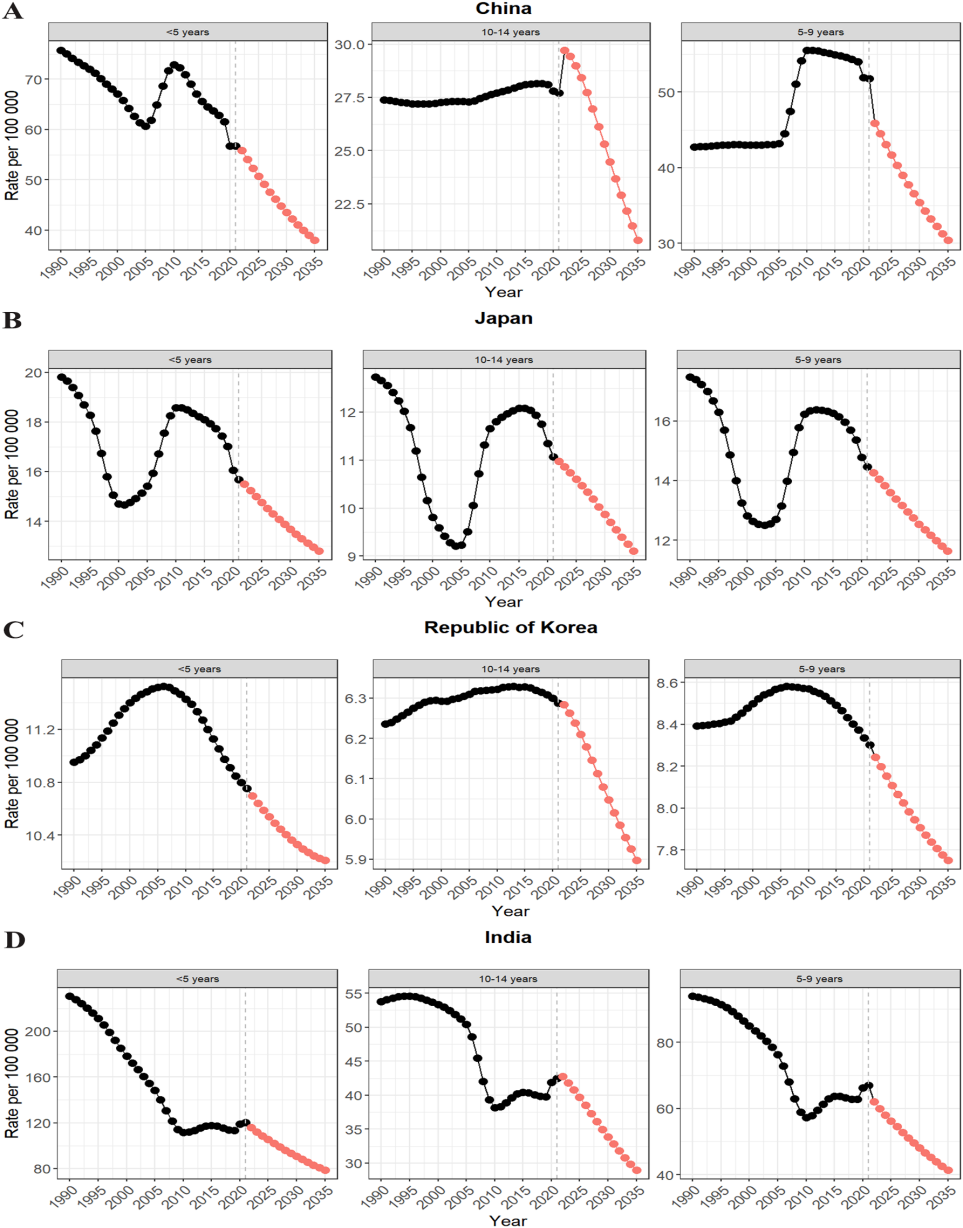
Projections of the burden (incidence) of encephalitis in children in China, Japan, Korea, Singapore and India to 2050. **(A)** China Incidence Rate Projections; **(B)** Japan Incidence Rate Projections; **(C)** Korea Incidence Rate Projections; **(D)** India Incidence Rate Projections.

In China, the incidence is projected to decrease to 28.993 (95% UI: 15.423–42.564) per 100,000, representing an approximately 41.33% reduction compared to 1990 (see **Figure 6A**).

Japan’s incidence is expected to drop to 10.951 (95% UI: 6.294–15.608) per 100,000, reflecting a decline of about 33.0% (see **Figure 6B**).

In South Korea, the incidence is projected to decrease to 7.880 (95% UI: 5.174–10.585) per 100,000, with a relatively smaller reduction of around 8.3% (see **Figure 6C**).

India is expected to experience the largest decline, with incidence falling to 49.105 (95% UI: 27.802–70.409) per 100,000, marking a decrease of approximately 62.4% compared to 1990 (see **Figure 6D**).

#### 3.6.2 Mortality prediction

**Figure 7:**
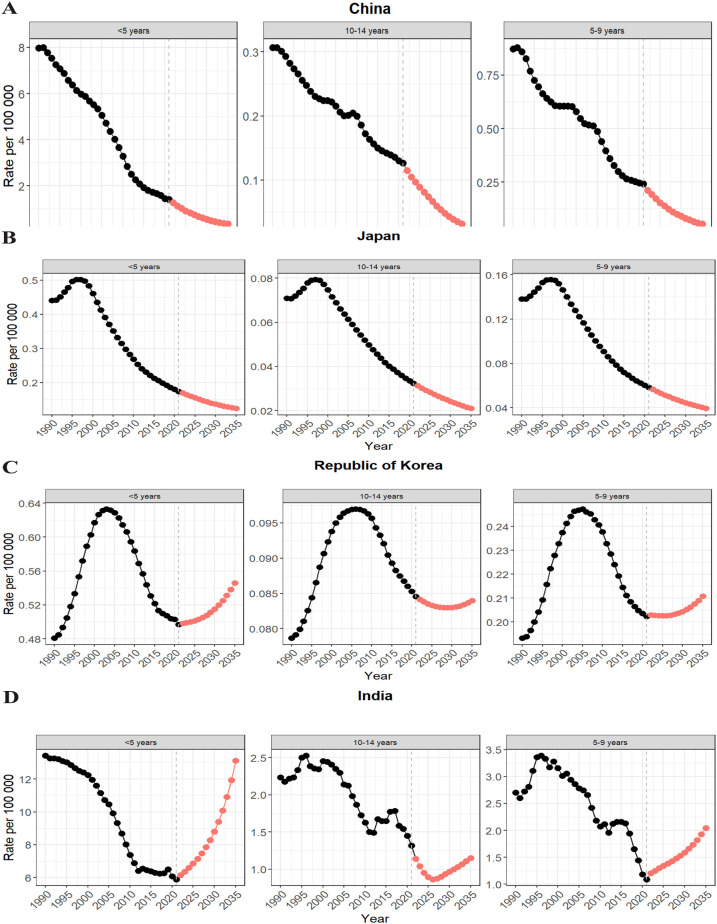
Projections of the burden (mortality) of encephalitis in children in China, Japan, Korea, Singapore and India to 2050. **(A)** China mortality rate projections; **(B)** Japan mortality rate projections; **(C)** Korea mortality rate projections; **(D)** India mortality rate projections.

Similarly, the mortality prediction results showed a downward trend. By 2035, the mortality rate for pediatric IE in China is expected to decline to 0.132 (95% UI: 0.018–0.246) per 100,000, representing a reduction of about 95.8% compared to 1990 (see **Figure 7A**).

Japan’s mortality rate is projected to decrease to 0.054 (95% UI: –0.0006–0.110) per 100,000, a decline of approximately 67.8% (see **Figure 7B**).

South Korea’s mortality rate is expected to drop to 0.253 (95% UI: 0.014–0.491) per 100,000, with a relatively modest reduction of only about 0.7%. (see **Figure 7C**) India’s mortality rate is forecasted to decrease to 4.10 (95% UI: –1.893–10.100) per 100,000, corresponding to a 62.4% reduction. (see **Figure 7D**) Overall, China is predicted to experience the most significant decrease in mortality, whereas South Korea will show the smallest reduction.

## 4 Discussion

This study utilized data from GBD to assess the incidence, mortality, and DALYs associated with IE among children aged 0–14 years in Asia from 1990 to 2021. It further compared the disease burden across age groups and regions, and examined the correlation between SDI and disease burden. Additionally, the study evaluated the risk factors for pediatric IE. Four representative Asian countries—China, Japan, South Korea, and India—were selected for further analysis, and the BAPC model was employed to project the epidemic trend of the disease through 2035. The findings indicated that although the incidence, fatality, and DALYs have significantly declined over time, IE remains a considerable public health challenge among children in Asia. In 2021, the number of cases exceeded 530,000, with nearly 20,000 deaths and approximately 1.7 million DALYs, with the highest burden observed in low-SDI regions such as Southeast and South Asia. These results aim to inform and guide current and future disease prevention and control efforts in relevant countries and regions.

Overall, the incidence, mortality, and DALYs associated with pediatric IE in Asia have demonstrated a marked downward trend, aligning with the global decline in the burden of most diseases. This trend is closely linked to sustained global investment in public health and continuous advancements in medical technology (Global incidence, prevalence, years lived with disability (YLDs), disability-adjusted life-years (DALYs), and healthy life expectancy (HALE) for 371 diseases and injuries in 204 countries and territories and 811 subnational locations, 1990-2021: a systematic analysis for the Global Burden of Disease Study 2021, 2024) In the clinical management of IE, the diagnosis in children is particularly challenging due to their atypical clinical manifestations, which heightens the risk of misdiagnosis or missed diagnosis of CNS infections. Etiological identification remains pivotal for effective treatment. In recent years, the widespread adoption of neuroimaging techniques such as cranial MRI and CT has facilitated precise localization of lesions, while advancements in pathogen detection technologies such as PCR and metagenomic next generation sequencing (NGS) have significantly improved both the detection rate and diagnostic accuracy (Piantadosi et al., 2021; Yang et al., 2022; Liu et al., 2024). These technological improvements have markedly enhanced early diagnosis and treatment, thereby contributing to the ongoing global and regional decline in incidence and mortality. Nevertheless, substantial disparities in disease burden persist among different countries and regions within Asia. South and Southeast Asia continue to exhibit high incidence and mortality rates, likely due to factors such as high population density, inadequate healthcare infrastructure, and suboptimal vaccination coverage (Wang et al., 2022). In contrast, high-income Asia-Pacific countries such as Japan, South Korea, Singapore demonstrated significantly lower incidence, mortality, and DALY rates. These nations benefit from higher SDI levels, robust public health and healthcare systems, and consistently high vaccination rates. Numerous global studies have affirmed the inverse relationship between SDI levels and disease burden (Cao et al., 2024; Kocarnik et al., 2022). Specifically, the negative correlation between SDI and the burden of IE is primarily influenced by the combined effects of education, income, and fertility. Higher levels of parental education heighten awareness of early neurological symptoms in children such as vomiting, headaches, and lethargy, which promotes timely medical consultation. Increased income facilitates access to higher-quality treatment and rehabilitation services, while lower fertility rates generally indicate greater per capita availability of maternal and child healthcare resources. Moreover, pediatric neurology is an exceptionally specialized field, and in regions with low SDI, the scarcity of qualified pediatric neurologists and healthcare professionals further exacerbates disparities. Therefore, promoting healthcare equity remains a crucial strategy for reducing the SDI gradient of childhood IE across Asia.

The COVID-19 pandemic may have had an impact on the epidemiology of infectious diseases. Therefore, we compared the incidence, mortality, and DALYs for infectious encephalitis between 2018 and 2021. The results revealed that the incidence rate in 2020 (49.24 per 100,000) was slightly higher than in 2019 (48.81 per 100,000), but still lower than in 2018 (49.32 per 100,000), suggesting that the COVID-19 pandemic had a minimal overall impact on the burden of IE. Furthermore, the mortality rate in 2020 (1.80 per 100,000) decreased compared to 2018 (1.98 per 100,000) and 2019 (1.95 per 100,000), reflecting a reduction in mortality. Similarly, the DALYs in 2020 decreased to 161.01 per 100,000, lower than in 2018 (176.95 per 100,000) and 2019 (174.30 per 100,000), indicating a decrease in disease burden. These findings suggest that, despite the pandemic’s impact on healthcare systems and surveillance, the disease burden of IE did not significantly decrease in 2020. The reduction in mortality and DALYs may be partially associated with enhanced public health measures, including reduced pathogen transmission. These results provide valuable insights into the stability of infectious encephalitis incidence during the pandemic period.

Notably, China, as a major developing nation in Asia, presents a unique profile: while it reports relatively high incidence rates of pediatric IE (see **Figure 3**), its mortality rate is substantially lower than that of South Asian countries such as India and Pakistan. This discrepancy may reflect improvements in China’s healthcare system, which have enhanced case detection rates, ensuring timely identification of infections (Li et al., 2023). Additionally, it suggests that China’s growing capacity to manage severe pediatric infectious diseases has contributed to the effective reduction of mortality (Tian et al., 2023).

Due to the lack of specific etiological data on IE in the GBD database, we were unable to directly analyze the composition and temporal trends of causative pathogens in pediatric IE. Nevertheless, existing studies on pediatric infectious meningitis provide valuable insights. A global study (Global, regional, and national burden of meningitis and its aetiologies, 1990-2019: a systematic analysis for the Global Burden of Disease Study 2019, 2023) conducted in 2019 revealed that among cases of infectious meningitis in children under five, viral pathogens accounted for 25.1%, *Neisseria meningitidis* for 16.9%, and *Streptococcus pneumoniae* for 13.3%. Among neonates, viruses comprised 37.1% of all cases, followed by Group B *Streptococcus* (GBS), which accounted for 20.4%. In terms of mortality, *Streptococcus pneumoniae* was the leading cause of meningitis-related deaths among children under five, responsible for 17.3% of fatalities. In neonates, Group B *Streptococcus* was the predominant fatal pathogen, accounting for as much as 22.8%. Given the clinical overlap and sequential occurrence of encephalitis and meningitis, it is reasonable to infer a degree of similarity in their etiological profiles within pediatric populations. Moreover, recent evidence indicates that pathogen evolution and advances in molecular diagnostics have significantly influenced the epidemiology of pediatric viral encephalitis. For example, the emergence of new enterovirus genotypes has altered regional epidemic patterns in East and South Asia (Xu et al., 2021). Meanwhile, the introduction of next-generation sequencing (NGS) and metagenomic approaches since 2010 has greatly improved pathogen identification, enabled the detection of previously unrecognized viral agents and enhanced case ascertainment accuracy (Pichler et al., 2025). These technological developments may partly explain regional differences in reported incidence and should be considered in interpreting temporal trends. The substantial reduction in the burden of pediatric IE across Asia over the past three decades can be largely attributed to the successful implementation of vaccination programs. Conjugate vaccines targeting *Haemophilus influenzae* type b (Hib), *Streptococcus pneumoniae*, and *Neisseria meningitidis* have played a pivotal role in reducing the incidence of severe bacterial infections in children (Wahl et al., 2018). However, unlike the major pathogens responsible for late-onset meningitis in children, there is currently no available vaccine for Group B *Streptococcus*. As a result, prevention of neonatal IE caused by GBS relies primarily on prenatal screening and intrapartum antibiotic prophylaxis (Steer et al., 2020). Therefore, the key to further reducing the burden of neonatal encephalitis lies in enhancing the accessibility, coverage, and standardization of maternal and perinatal healthcare services.

Our Study shown sex-based disparities in IE: the incidence is significantly higher in boys, while girls exhibit slightly higher mortality rates and DALYs. Some research (Dharmarajan et al., 2016; Peer et al., 2019) has documented gender differences in the epidemiology and clinical outcomes of pediatric CNS infections; however, the underlying pathophysiological mechanisms remain insufficiently explored. These disparities may be attributed to differences in immune responses and physiological structures, but further mechanistic studies are needed to substantiate these hypotheses (Muenchhoff and Goulder, 2014; McCarthy, 2019).From an age-structured perspective, there is a generally inverse relationship between age and the burden of IE. Younger children, particularly those aged 0–4 years, bear the heaviest burden, with markedly higher incidence, mortality, and DALY rates compared to older age groups. This finding aligns with conclusions from other studies on age-specific CNS infections caused by various pathogens (Basatemur, 2023; Liu et al., 2025; Peng et al., 2025). The disproportionate burden in this age group is likely associated with the immaturity of the immune system, underdeveloped blood-brain barrier, and atypical clinical manifestations in infants and young children.

Malnutrition and prematurity have long been recognized as major risk factors for numerous diseases, particularly in low-income countries across Africa, Asia, and Latin America (Black et al., 2013; Keats et al., 2021). Our analysis similarly identified malnutrition and prematurity as primary risk factors for IE. In addition, this study found that particulate matter pollution also constitutes a significant environmental risk factor for IE. These findings suggest that future encephalitis prevention strategies in Asia should prioritize environmental improvement and nutritional interventions.

To project future trends in the burden of pediatric IE in representative Asian countries, we employed BAPC model. Our forecasts indicated that the burden of IE in China, India, Japan, and South Korea will continue to decline over the next decade, with particularly pronounced reductions in mortality and DALYs. However, it is important to note that while middle- and low-income countries such as India are projected to experience a downward trend, their overall burden is expected to remain relatively high. This underscores the continued need for strengthened disease control measures and expanded vaccination efforts. In contrast, high-income countries such as Japan and South Korea, despite maintaining a low disease burden, are also projected to experience further declines. Nevertheless, these countries may face emerging challenges in the prevention and control of pediatric IE due to stagnant economic growth, declining birth rates, and the increasing pressures of healthcare resource reallocation in aging societies.

This study has several limitations. First, the data were primarily derived from GBD database, which reflects not only the healthcare management capacity of individual countries but also their ability to collect and report data. In regions affected by conflict or political instability, data collection may be compromised, potentially leading to substantial inaccuracies or underestimation of disease burden. Second, etiological analysis plays a crucial role in informing prevention strategies for encephalitis, and the distribution of pathogens may vary over time. However, the GBD database currently lacks detailed pathogen-specific data for pediatric IE. Lastly, although our use of the BAPC model provides prospective insights, the predictive results are inherently limited by the assumptions of the model. The emergence of novel pathogens or large-scale public health events, such as the COVID-19 pandemic, could significantly impact the accuracy of these forecasts.

## 5 Conclusion

Between 1990 and 2021, the burden of infectious IE among children in Asia declined significantly, with notable reductions in incidence, mortality, and disability-adjusted life years (DALYs). However, substantial disparities remain across regions and countries. Socioeconomically underdeveloped areas, such as South Asia, continue to experience a high disease burden, whereas high-income regions in the Asia-Pacific report relatively low impact. Targeted interventions are recommended in high-burden areas, including strengthening perinatal care to reduce low birth weight and preterm births, improving air quality to lower environmental exposure risks, and promoting vaccination programs along with early diagnosis and treatment strategies.

## Data Availability

Publicly available datasets were analyzed in this study. This data can be found here: https://ghdx.healthdata.org/gbd-2021.
