# Supplementary material for: The burden of infective encephalitis in children in Asian countries (1990–2021): systematic analysis and projection of the burden of disease
Source: Front Cell Infect Microbiol. 2025 Nov 20;15:1682224. doi: 10.3389/fcimb.2025.1682224 (PMC12675410; doi:10.3389/fcimb.2025.1682224)
Supplement: Supplementary file 1 [file Table1.docx]

**Supplementary table 1.** Incidence rate of encephalitis among male children in Asian countries and regions from 1990 to 2021.

| **level** | **1990** | | **2021** | | **1990–2021** | |
| --- | --- | --- | --- | --- | --- | --- |
|  | **Number (95 %UI)** | **Rate per 100,000(95%UI)** | **Number (95 %UI)** | **Rate per 100,000(95%UI)** | **Rate change** | **Rate EAPC(95 %CI)** |
| Afghanistan | 433.634(367.839-518.577) | 19.742(16.747-23.610) | 1326.973(1113.838-1574.194) | 18.023(15.128-21.381) | -0.087(-0.160--0.010) | -0.53(-0.62,-0.45) |
| Algeria | 616.227(480.386-764.978) | 11.234(8.758-13.946) | 706.393(560.177-898.682) | 10.329(8.191-13.141) | -0.081(-0.147--0.003) | -0.06(-0.16,0.03) |
| Armenia | 82.003(65.947-102.870) | 15.319(12.320-19.218) | 43.612(34.830-54.638) | 13.889(11.092-17.400) | -0.093(-0.174--0.009) | -0.12(-0.24,-0.00) |
| Azerbaijan | 297.228(255.160-350.909) | 23.847(20.471-28.153) | 255.917(220.445-302.030) | 20.306(17.491-23.964) | -0.148(-0.215--0.072) | -0.24(-0.37,-0.10) |
| Bahrain | 9.546(7.420-12.019) | 11.392(8.856-14.343) | 15.204(11.915-19.701) | 9.970(7.814-12.920) | -0.125(-0.196--0.044) | -0.36(-0.43,-0.29) |
| Bangladesh | 16941.914(13599.561-21183.628) | 67.530(54.208-84.438) | 12532.004(10100.855-15667.745) | 53.856(43.408-67.332) | -0.202(-0.274--0.122) | -0.63(-0.70,-0.57) |
| Bhutan | 111.372(95.057-131.051) | 81.948(69.943-96.427) | 74.819(64.113-87.130) | 79.345(67.992-92.401) | -0.032(-0.129-0.065) | -0.17(-0.28,-0.07) |
| Brunei Darussalam | 5.074(4.053-6.551) | 10.825(8.648-13.976) | 5.616(4.479-6.935) | 11.385(9.080-14.058) | 0.052(-0.051-0.152) | 0.16(0.11,0.22) |
| Cambodia | 672.678(556.267-804.674) | 28.637(23.681-34.256) | 530.735(431.112-638.537) | 20.245(16.445-24.357) | -0.293(-0.364--0.213) | -1.15(-1.19,-1.12) |
| China | 89009.955(74619.364-106589.897) | 53.523(44.870-64.094) | 69481.330(57387.484-84016.757) | 50.143(41.415-60.633) | -0.063(-0.138-0.009) | 0.31(0.03,0.59) |
| Democratic People's Republic of Korea | 1196.433(985.705-1445.586) | 39.819(32.805-48.111) | 766.335(627.544-941.044) | 31.378(25.695-38.532) | -0.212(-0.287--0.126) | -0.82(-0.88,-0.76) |
| Egypt | 1559.983(1298.833-1911.001) | 13.657(11.370-16.729) | 1915.473(1499.648-2444.064) | 10.055(7.873-12.830) | -0.264(-0.337--0.189) | -0.86(-0.94,-0.79) |
| Georgia | 112.959(89.481-140.777) | 16.188(12.823-20.174) | 52.611(44.571-62.496) | 13.688(11.596-16.259) | -0.154(-0.259--0.038) | -0.47(-0.56,-0.38) |
| India | 203753.477(177564.107-235965.630) | 119.890(104.480-138.844) | 141336.032(122231.539-164643.449) | 73.722(63.757-85.879) | -0.385(-0.414--0.355) | -2.07(-2.33,-1.80) |
| Indonesia | 7270.729(5632.316-9395.236) | 20.932(16.215-27.048) | 4802.670(3898.247-6057.572) | 13.909(11.290-17.543) | -0.336(-0.368--0.295) | -1.88(-2.16,-1.60) |
| Iraq | 912.119(781.180-1069.257) | 21.551(18.457-25.264) | 1131.351(949.090-1368.048) | 16.321(13.692-19.736) | -0.243(-0.315--0.167) | -0.87(-0.98,-0.75) |
| Islamic Republic of Iran | 1422.500(1129.681-1815.957) | 10.996(8.733-14.038) | 1002.361(788.514-1265.007) | 9.674(7.610-12.209) | -0.120(-0.149--0.093) | -0.04(-0.18,0.10) |
| Japan | 1996.399(1518.548-2607.549) | 16.863(12.827-22.025) | 1112.564(834.627-1469.241) | 14.024(10.520-18.520) | -0.168(-0.197--0.144) | 0.04(-0.38,0.46) |
| Jordan | 96.454(75.756-123.052) | 11.497(9.030-14.667) | 182.242(140.057-233.088) | 9.761(7.502-12.485) | -0.151(-0.234--0.075) | -0.51(-0.61,-0.40) |
| Kazakhstan | 758.465(663.033-873.950) | 28.805(25.181-33.191) | 573.869(497.816-666.681) | 20.591(17.862-23.922) | -0.285(-0.344--0.230) | -1.40(-1.64,-1.17) |
| Kuwait | 32.347(25.589-41.510) | 11.461(9.067-14.708) | 44.265(34.768-56.304) | 10.187(8.001-12.958) | -0.111(-0.189--0.001) | -0.27(-0.37,-0.18) |
| Kyrgyzstan | 215.059(183.749-251.144) | 25.369(21.676-29.626) | 181.845(150.606-220.964) | 15.622(12.938-18.982) | -0.384(-0.452--0.315) | -1.67(-1.82,-1.53) |
| Lao People's Democratic Republic | 237.696(197.678-287.832) | 25.459(21.173-30.829) | 238.989(197.887-288.352) | 20.440(16.924-24.661) | -0.197(-0.273--0.120) | -0.78(-0.82,-0.74) |
| Lebanon | 64.689(52.304-82.407) | 11.899(9.621-15.158) | 70.887(56.322-90.019) | 10.595(8.418-13.454) | -0.110(-0.201--0.019) | -0.22(-0.30,-0.14) |
| Libya | 80.463(63.688-102.503) | 8.928(7.066-11.373) | 60.951(46.883-78.396) | 7.981(6.139-10.266) | -0.106(-0.183--0.013) | -0.32(-0.38,-0.25) |
| Malaysia | 847.402(681.623-1036.727) | 25.147(20.227-30.765) | 865.139(684.399-1066.217) | 22.062(17.453-27.189) | -0.123(-0.190--0.046) | -0.46(-0.49,-0.42) |
| Maldives | 14.438(11.867-17.968) | 26.998(22.191-33.600) | 9.481(7.472-11.813) | 18.373(14.480-22.893) | -0.319(-0.397--0.220) | -1.12(-1.32,-0.93) |
| Mauritius | 34.063(27.118-43.400) | 20.378(16.223-25.964) | 24.742(19.953-30.134) | 23.554(18.995-28.688) | 0.156(0.033-0.288) | 0.56(0.53,0.60) |
| Mongolia | 145.650(128.582-168.431) | 32.047(28.291-37.059) | 125.956(109.569-145.408) | 22.630(19.686-26.126) | -0.294(-0.354--0.231) | -0.75(-0.90,-0.59) |
| Morocco | 553.786(444.525-696.905) | 11.196(8.987-14.089) | 509.410(407.900-637.736) | 10.148(8.126-12.705) | -0.094(-0.165--0.019) | -0.24(-0.32,-0.17) |
| Myanmar | 1719.577(1416.836-2126.906) | 23.065(19.005-28.529) | 1627.872(1357.576-1978.037) | 20.495(17.092-24.903) | -0.111(-0.185--0.035) | -0.36(-0.52,-0.19) |
| Nepal | 3820.045(3214.717-4541.471) | 88.550(74.518-105.273) | 2615.951(2257.141-3098.931) | 55.259(47.680-65.461) | -0.376(-0.439--0.307) | -1.97(-2.17,-1.76) |
| Oman | 116.798(100.574-137.140) | 27.273(23.485-32.023) | 102.341(85.687-124.389) | 16.405(13.735-19.939) | -0.398(-0.451--0.335) | -1.64(-1.66,-1.61) |
| Pakistan | 23389.317(20216.889-27220.466) | 91.428(79.027-106.404) | 45259.849(38741.691-52725.718) | 102.148(87.437-118.998) | 0.117(0.054-0.192) | 0.47(0.41,0.53) |
| Palestine | 58.655(47.255-73.335) | 11.783(9.493-14.732) | 99.980(77.295-127.136) | 10.446(8.076-13.283) | -0.114(-0.194--0.028) | -0.34(-0.44,-0.25) |
| Philippines | 4710.558(3891.920-5582.004) | 36.465(30.128-43.211) | 5977.538(4996.315-7052.805) | 33.975(28.398-40.087) | -0.068(-0.098--0.039) | -0.22(-0.36,-0.08) |
| Qatar | 7.980(6.484-9.868) | 12.381(10.059-15.310) | 26.838(21.394-33.873) | 10.662(8.499-13.457) | -0.139(-0.208--0.057) | -0.33(-0.42,-0.25) |
| Republic of Korea | 518.393(386.860-680.683) | 8.779(6.552-11.528) | 270.474(204.375-356.442) | 8.703(6.576-11.469) | -0.009(-0.094-0.085) | -0.06(-0.14,0.01) |
| Saudi Arabia | 392.913(308.409-490.840) | 11.760(9.230-14.691) | 384.306(298.029-503.846) | 9.685(7.511-12.697) | -0.176(-0.250--0.106) | -0.64(-0.65,-0.62) |
| Seychelles | 2.408(1.920-3.096) | 19.982(15.929-25.689) | 2.278(1.802-2.828) | 19.148(15.146-23.778) | -0.042(-0.129-0.048) | -0.06(-0.13,0.01) |
| Singapore | 15.832(12.727-19.549) | 4.702(3.780-5.807) | 19.573(15.386-24.798) | 4.801(3.774-6.083) | 0.021(-0.062-0.112) | -0.27(-0.99,0.45) |
| Socialist Republic of Viet Nam | 6407.820(5478.387-7619.445) | 47.220(40.371-56.148) | 5253.655(4480.865-6176.045) | 40.726(34.735-47.876) | -0.138(-0.210--0.037) | -0.36(-0.42,-0.30) |
| Sri Lanka | 764.403(628.984-932.817) | 27.269(22.438-33.277) | 593.398(478.852-717.891) | 22.943(18.514-27.756) | -0.159(-0.232--0.085) | -0.49(-0.53,-0.44) |
| Sudan | 538.235(437.805-679.882) | 11.659(9.483-14.727) | 874.356(706.709-1093.958) | 10.202(8.246-12.764) | -0.125(-0.198--0.040) | -0.45(-0.50,-0.39) |
| Syrian Arab Republic | 397.880(322.647-493.885) | 13.117(10.637-16.283) | 193.902(154.599-244.301) | 10.395(8.288-13.097) | -0.208(-0.282--0.128) | -0.91(-1.00,-0.83) |
| Taiwan (Province of China) | 605.159(474.387-765.369) | 21.286(16.686-26.921) | 226.887(180.644-284.731) | 14.843(11.818-18.627) | -0.303(-0.359--0.239) | -1.31(-1.57,-1.04) |
| Tajikistan | 192.120(152.732-244.594) | 16.317(12.971-20.773) | 249.672(195.016-317.466) | 13.488(10.535-17.150) | -0.173(-0.237--0.102) | -0.48(-0.60,-0.37) |
| Thailand | 2909.939(2423.982-3484.394) | 33.871(28.215-40.558) | 968.955(763.960-1199.778) | 19.323(15.235-23.927) | -0.430(-0.491--0.357) | -2.25(-2.41,-2.08) |
| Timor-Leste | 53.849(45.414-64.461) | 31.099(26.228-37.228) | 62.751(52.146-75.551) | 23.357(19.409-28.121) | -0.249(-0.320--0.169) | -1.13(-1.20,-1.05) |
| Tunisia | 180.650(143.638-232.257) | 11.361(9.033-14.607) | 148.332(117.064-185.974) | 10.300(8.128-12.913) | -0.093(-0.168--0.014) | -0.14(-0.21,-0.06) |
| Turkey | 1421.329(1129.356-1770.797) | 13.503(10.729-16.823) | 1102.089(863.319-1414.315) | 11.593(9.081-14.878) | -0.141(-0.219--0.049) | -0.70(-0.83,-0.57) |
| Turkmenistan | 136.296(114.246-162.945) | 17.943(15.040-21.451) | 184.510(161.793-212.867) | 23.539(20.641-27.156) | 0.312(0.185-0.457) | 1.07(0.84,1.29) |
| United Arab Emirates | 38.224(30.532-48.099) | 12.621(10.081-15.881) | 75.688(59.716-94.956) | 11.042(8.712-13.853) | -0.125(-0.199--0.049) | -0.18(-0.26,-0.10) |
| Uzbekistan | 1243.215(1099.246-1426.768) | 28.713(25.388-32.952) | 1327.023(1163.934-1515.518) | 25.365(22.248-28.968) | -0.117(-0.186--0.043) | -0.08(-0.22,0.05) |
| Yemen | 421.163(336.950-520.642) | 11.405(9.125-14.099) | 726.919(590.180-906.363) | 10.268(8.336-12.803) | -0.100(-0.172--0.005) | -0.21(-0.36,-0.06) |

**Supplementary table 2:** Incidence rate of encephalitis among female children in Asian countries and regions from 1990 to 2021.

| **level** | **1990** | | **2021** | | **1990–2021** | |
| --- | --- | --- | --- | --- | --- | --- |
|  | **Number (95 %UI)** | **Rate per 100,000(95%UI)** | **Number (95 %UI)** | **Rate per 100,000(95%UI)** | **Rate change** | **Rate EAPC(95 %CI)** |
| Afghanistan | 459.027(388.953-549.976) | 21.738(18.420-26.045) | 1318.719(1139.098-1561.006) | 19.285(16.658-22.829) | -0.113(-0.186--0.042) | -0.59(-0.68,-0.50) |
| Algeria | 649.061(520.076-812.807) | 12.388(9.927-15.514) | 701.975(554.679-892.933) | 10.861(8.582-13.816) | -0.123(-0.199--0.034) | -0.22(-0.31,-0.12) |
| Armenia | 71.444(58.084-89.531) | 14.066(11.436-17.627) | 34.314(27.470-43.703) | 12.327(9.869-15.700) | -0.124(-0.194--0.042) | -0.16(-0.30,-0.02) |
| Azerbaijan | 359.661(315.339-412.312) | 30.466(26.712-34.926) | 295.799(259.722-339.579) | 26.884(23.606-30.864) | -0.118(-0.187--0.048) | 0.10(-0.10,0.30) |
| Bahrain | 9.627(7.602-12.043) | 12.117(9.568-15.158) | 15.527(12.044-19.857) | 10.766(8.351-13.768) | -0.111(-0.191--0.032) | -0.28(-0.34,-0.22) |
| Bangladesh | 15082.297(12147.270-18776.272) | 63.308(50.988-78.814) | 10660.635(8377.022-13280.465) | 47.388(37.237-59.033) | -0.251(-0.319--0.168) | -0.84(-0.94,-0.73) |
| Bhutan | 104.421(88.874-124.126) | 82.685(70.374-98.288) | 65.474(55.955-76.453) | 70.500(60.251-82.321) | -0.147(-0.227--0.068) | -0.62(-0.69,-0.54) |
| Brunei Darussalam | 3.669(2.862-4.798) | 8.394(6.548-10.977) | 3.681(2.841-4.850) | 8.131(6.277-10.715) | -0.031(-0.126-0.073) | -0.12(-0.15,-0.09) |
| Cambodia | 494.737(411.671-598.131) | 21.399(17.806-25.871) | 377.394(306.006-463.997) | 15.126(12.265-18.597) | -0.293(-0.365--0.224) | -1.20(-1.23,-1.17) |
| China | 68348.467(56866.698-82882.487) | 44.944(37.394-54.501) | 48040.601(39193.416-59079.568) | 39.681(32.374-48.800) | -0.117(-0.186--0.049) | 0.10(-0.17,0.38) |
| Democratic People's Republic of Korea | 1053.522(881.229-1270.103) | 35.779(29.927-43.134) | 616.596(507.484-765.031) | 26.447(21.767-32.813) | -0.261(-0.330--0.188) | -1.08(-1.14,-1.02) |
| Egypt | 1516.267(1236.554-1840.786) | 14.093(11.493-17.110) | 1876.657(1467.422-2424.807) | 10.539(8.241-13.618) | -0.252(-0.333--0.166) | -0.86(-0.92,-0.80) |
| Georgia | 92.764(73.080-114.902) | 13.827(10.893-17.126) | 38.243(32.059-45.245) | 10.878(9.119-12.869) | -0.213(-0.298--0.100) | -0.68(-0.83,-0.52) |
| India | 223125.025(194735.944-254564.032) | 142.508(124.376-162.587) | 131096.389(113715.834-151426.860) | 75.048(65.098-86.686) | -0.473(-0.502--0.442) | -2.65(-2.98,-2.32) |
| Indonesia | 7302.435(5684.985-9493.811) | 22.127(17.226-28.767) | 4316.150(3498.337-5429.987) | 13.175(10.679-16.575) | -0.405(-0.435--0.369) | -2.29(-2.60,-1.99) |
| Iraq | 998.401(852.037-1178.238) | 24.934(21.279-29.426) | 1234.978(1026.933-1468.197) | 18.912(15.726-22.483) | -0.242(-0.309--0.173) | -0.95(-1.11,-0.79) |
| Islamic Republic of Iran | 1498.532(1194.215-1925.925) | 12.038(9.593-15.471) | 1006.937(791.270-1291.650) | 10.256(8.059-13.156) | -0.148(-0.182--0.116) | -0.16(-0.30,-0.02) |
| Japan | 1777.491(1347.635-2304.224) | 15.800(11.979-20.482) | 976.485(734.124-1283.506) | 13.001(9.774-17.089) | -0.177(-0.205--0.150) | -0.08(-0.47,0.30) |
| Jordan | 108.189(87.851-133.508) | 13.618(11.058-16.805) | 188.154(148.930-245.441) | 10.654(8.433-13.898) | -0.218(-0.294--0.134) | -0.91(-1.06,-0.77) |
| Kazakhstan | 655.051(567.219-755.428) | 25.557(22.130-29.473) | 465.318(394.749-545.510) | 17.629(14.955-20.667) | -0.310(-0.372--0.245) | -1.67(-1.92,-1.42) |
| Kuwait | 34.682(26.958-44.024) | 12.745(9.907-16.178) | 44.651(34.655-56.686) | 10.865(8.433-13.793) | -0.148(-0.225--0.067) | -0.35(-0.48,-0.22) |
| Kyrgyzstan | 167.801(142.475-198.422) | 20.220(17.169-23.910) | 148.187(120.933-179.721) | 13.346(10.892-16.186) | -0.340(-0.409--0.267) | -1.36(-1.50,-1.23) |
| Lao People's Democratic Republic | 183.254(150.069-221.483) | 20.150(16.501-24.353) | 172.409(141.083-211.749) | 15.296(12.517-18.787) | -0.241(-0.316--0.165) | -0.99(-1.05,-0.92) |
| Lebanon | 63.492(50.470-80.301) | 12.642(10.050-15.989) | 68.246(53.577-87.694) | 11.207(8.798-14.400) | -0.114(-0.194--0.040) | -0.27(-0.35,-0.20) |
| Libya | 85.280(66.727-109.635) | 9.375(7.335-12.052) | 60.122(47.064-77.226) | 8.259(6.465-10.609) | -0.119(-0.192--0.038) | -0.33(-0.41,-0.26) |
| Malaysia | 631.723(506.695-794.531) | 19.722(15.819-24.805) | 628.463(495.986-792.195) | 17.021(13.433-21.456) | -0.137(-0.212--0.049) | -0.49(-0.52,-0.46) |
| Maldives | 10.927(9.057-13.463) | 21.191(17.564-26.110) | 6.728(5.372-8.405) | 13.853(11.061-17.306) | -0.346(-0.421--0.265) | -1.20(-1.40,-1.00) |
| Mauritius | 28.401(22.908-34.912) | 17.434(14.063-21.432) | 17.580(14.049-21.793) | 17.174(13.725-21.290) | -0.015(-0.098-0.074) | -0.19(-0.24,-0.15) |
| Mongolia | 94.458(81.128-113.331) | 21.202(18.210-25.438) | 83.532(71.132-98.253) | 15.760(13.420-18.537) | -0.257(-0.323--0.186) | -0.52(-0.71,-0.33) |
| Morocco | 595.574(467.631-768.213) | 12.307(9.663-15.874) | 535.225(416.224-680.848) | 11.215(8.722-14.267) | -0.089(-0.180--0.007) | -0.21(-0.30,-0.12) |
| Myanmar | 1356.340(1099.897-1685.114) | 18.526(15.024-23.017) | 1102.204(891.115-1345.649) | 14.367(11.615-17.540) | -0.225(-0.296--0.149) | -0.89(-0.97,-0.81) |
| Nepal | 3396.512(2911.086-3972.429) | 82.617(70.809-96.625) | 2264.995(1945.877-2657.880) | 50.409(43.307-59.153) | -0.390(-0.452--0.333) | -1.95(-2.10,-1.80) |
| Oman | 110.424(93.616-131.332) | 26.794(22.716-31.867) | 82.182(66.966-102.634) | 13.716(11.176-17.129) | -0.488(-0.545--0.431) | -2.34(-2.46,-2.21) |
| Pakistan | 12767.303(10578.559-15666.904) | 53.962(44.711-66.217) | 17414.419(13960.435-21199.395) | 42.336(33.939-51.538) | -0.215(-0.258--0.170) | -0.55(-0.66,-0.45) |
| Palestine | 59.802(48.131-75.543) | 12.710(10.230-16.056) | 101.360(80.360-127.172) | 11.138(8.830-13.974) | -0.124(-0.204--0.039) | -0.31(-0.40,-0.22) |
| Philippines | 3645.328(3028.114-4354.098) | 29.646(24.626-35.410) | 4553.957(3804.313-5426.960) | 27.760(23.190-33.082) | -0.064(-0.104--0.024) | -0.11(-0.23,0.01) |
| Qatar | 9.258(7.613-11.406) | 15.280(12.566-18.825) | 27.850(22.003-35.030) | 11.499(9.085-14.464) | -0.247(-0.311--0.180) | -0.89(-0.94,-0.84) |
| Republic of Korea | 419.584(314.423-550.457) | 7.676(5.752-10.071) | 225.319(170.726-297.554) | 7.595(5.755-10.030) | -0.011(-0.117-0.076) | -0.05(-0.13,0.04) |
| Saudi Arabia | 473.953(385.874-587.399) | 14.755(12.013-18.287) | 386.970(301.840-501.549) | 10.758(8.391-13.943) | -0.271(-0.348--0.191) | -1.04(-1.11,-0.97) |
| Seychelles | 1.908(1.525-2.413) | 16.343(13.059-20.666) | 1.736(1.364-2.189) | 15.079(11.848-19.014) | -0.077(-0.158-0.014) | -0.19(-0.26,-0.11) |
| Singapore | 13.512(10.783-16.735) | 4.321(3.448-5.352) | 17.264(13.805-21.647) | 4.268(3.413-5.352) | -0.012(-0.092-0.076) | -0.12(-0.86,0.63) |
| Socialist Republic of Viet Nam | 3677.573(3124.914-4438.413) | 28.415(24.145-34.294) | 2546.941(2128.054-3031.040) | 21.471(17.939-25.551) | -0.244(-0.316--0.171) | -0.73(-0.85,-0.62) |
| Sri Lanka | 590.301(484.174-725.327) | 21.624(17.736-26.571) | 424.158(335.450-529.692) | 16.848(13.324-21.040) | -0.221(-0.296--0.148) | -0.76(-0.82,-0.71) |
| Sudan | 573.918(465.012-704.182) | 13.422(10.875-16.469) | 851.928(679.282-1075.959) | 10.624(8.471-13.418) | -0.208(-0.287--0.109) | -0.75(-0.82,-0.67) |
| Syrian Arab Republic | 416.558(338.270-516.507) | 14.419(11.709-17.879) | 194.839(154.348-246.345) | 10.836(8.584-13.701) | -0.248(-0.318--0.175) | -1.12(-1.21,-1.02) |
| Taiwan (Province of China) | 475.719(373.041-612.198) | 17.850(13.997-22.971) | 187.282(149.665-236.103) | 13.206(10.554-16.649) | -0.260(-0.320--0.200) | -1.14(-1.38,-0.90) |
| Tajikistan | 171.133(136.692-214.058) | 14.951(11.942-18.702) | 220.135(172.446-279.226) | 12.701(9.949-16.110) | -0.151(-0.221--0.054) | -0.35(-0.46,-0.23) |
| Thailand | 1702.354(1389.433-2084.309) | 20.591(16.806-25.211) | 747.925(589.389-939.056) | 15.739(12.403-19.762) | -0.236(-0.311--0.150) | -1.03(-1.08,-0.98) |
| Timor-Leste | 40.658(34.152-49.591) | 25.494(21.415-31.096) | 46.059(38.077-55.819) | 18.279(15.111-22.152) | -0.283(-0.350--0.206) | -1.37(-1.48,-1.25) |
| Tunisia | 186.667(148.183-234.222) | 12.318(9.778-15.456) | 145.080(114.882-187.142) | 10.945(8.667-14.118) | -0.111(-0.176--0.038) | -0.18(-0.27,-0.10) |
| Turkey | 1692.579(1368.285-2103.018) | 16.989(13.734-21.109) | 1182.809(923.563-1527.886) | 13.122(10.246-16.950) | -0.228(-0.311--0.149) | -1.02(-1.15,-0.90) |
| Turkmenistan | 117.737(98.276-145.052) | 15.884(13.258-19.569) | 167.160(147.014-189.624) | 22.585(19.863-25.620) | 0.422(0.276-0.584) | 1.31(1.03,1.60) |
| United Arab Emirates | 37.826(30.394-47.170) | 13.202(10.608-16.463) | 74.311(57.140-94.433) | 11.374(8.746-14.454) | -0.138(-0.221--0.047) | -0.23(-0.32,-0.15) |
| Uzbekistan | 1036.704(902.239-1199.968) | 24.533(21.351-28.396) | 924.460(790.857-1065.233) | 19.024(16.275-21.921) | -0.225(-0.290--0.152) | -0.66(-0.75,-0.58) |
| Yemen | 448.666(367.143-555.623) | 13.190(10.793-16.334) | 731.527(584.439-913.462) | 10.904(8.711-13.615) | -0.173(-0.258--0.083) | -0.45(-0.60,-0.31) |

**Supplementary table 3:** Incidence rate of encephalitis among children in Asian countries and regions from 1990 to 2021.

| **level** | **1990** | | **2021** | | **1990–2021** | |
| --- | --- | --- | --- | --- | --- | --- |
|  | **Number (95 %UI)** | **Rate per 100,000(95%UI)** | **Number (95 %UI)** | **Rate per 100,000(95%UI)** | **Rate change** | **Rate EAPC(95 %CI)** |
| Afghanistan | 892.661(764.117-1067.395) | 20.721(17.737-24.777) | 2645.693(2259.007-3122.489) | 18.631(15.908-21.988) | -0.101(-0.154--0.044) | -0.56(-0.65,-0.48) |
| Algeria | 1265.288(1005.018-1580.942) | 11.798(9.371-14.741) | 1408.368(1123.006-1780.784) | 10.588(8.443-13.388) | -0.103(-0.158--0.049) | -0.14(-0.24,-0.05) |
| Armenia | 153.447(125.395-190.785) | 14.709(12.020-18.288) | 77.927(62.145-97.815) | 13.155(10.491-16.513) | -0.106(-0.168--0.048) | -0.13(-0.26,0.00) |
| Azerbaijan | 656.890(576.447-758.213) | 27.067(23.752-31.242) | 551.715(486.034-630.692) | 23.372(20.590-26.718) | -0.137(-0.188--0.082) | -0.08(-0.24,0.09) |
| Bahrain | 19.174(15.040-23.947) | 11.745(9.213-14.669) | 30.731(24.074-39.685) | 10.357(8.114-13.375) | -0.118(-0.173--0.061) | -0.32(-0.39,-0.26) |
| Bangladesh | 32024.211(25842.225-40034.116) | 65.474(52.835-81.850) | 23192.640(18514.043-28947.976) | 50.677(40.454-63.252) | -0.226(-0.276--0.170) | -0.73(-0.80,-0.66) |
| Bhutan | 215.793(185.227-254.193) | 82.303(70.645-96.948) | 140.293(121.236-162.737) | 74.957(64.775-86.948) | -0.089(-0.162--0.016) | -0.39(-0.47,-0.30) |
| Brunei Darussalam | 8.742(6.866-11.287) | 9.652(7.580-12.461) | 9.297(7.406-11.592) | 9.828(7.828-12.254) | 0.018(-0.049-0.090) | 0.06(0.03,0.08) |
| Cambodia | 1167.415(975.481-1404.919) | 25.047(20.929-30.142) | 908.129(739.292-1090.344) | 17.749(14.449-21.310) | -0.291(-0.347--0.236) | -1.17(-1.19,-1.14) |
| China | 157358.422(131637.980-189155.169) | 49.425(41.347-59.412) | 117521.931(96364.106-142760.853) | 45.265(37.116-54.986) | -0.084(-0.157--0.018) | 0.23(-0.05,0.51) |
| Democratic People's Republic of Korea | 2249.955(1881.392-2708.928) | 37.819(31.624-45.534) | 1382.931(1131.547-1696.810) | 28.970(23.704-35.545) | -0.234(-0.291--0.164) | -0.94(-0.99,-0.88) |
| Egypt | 3076.250(2543.438-3736.291) | 13.868(11.466-16.844) | 3792.130(2982.018-4855.251) | 10.289(8.091-13.174) | -0.258(-0.325--0.185) | -0.86(-0.93,-0.79) |
| Georgia | 205.724(164.096-257.483) | 15.030(11.989-18.812) | 90.854(77.326-107.279) | 12.345(10.507-14.577) | -0.179(-0.266--0.077) | -0.54(-0.66,-0.43) |
| India | 426878.502(372477.870-491065.115) | 130.735(114.075-150.393) | 272432.421(236255.864-316191.389) | 74.354(64.480-86.297) | -0.431(-0.458--0.404) | -2.36(-2.66,-2.06) |
| Indonesia | 14573.164(11316.531-18905.073) | 21.514(16.707-27.909) | 9118.820(7394.195-11517.280) | 13.552(10.989-17.116) | -0.370(-0.397--0.334) | -2.08(-2.37,-1.79) |
| Iraq | 1910.520(1650.037-2214.088) | 23.196(20.033-26.882) | 2366.329(1988.802-2794.538) | 17.578(14.774-20.759) | -0.242(-0.294--0.188) | -0.91(-1.05,-0.77) |
| Islamic Republic of Iran | 2921.032(2321.070-3756.244) | 11.507(9.144-14.797) | 2009.298(1573.876-2562.919) | 9.957(7.799-12.701) | -0.135(-0.163--0.105) | -0.10(-0.24,0.04) |
| Japan | 3773.889(2868.212-4906.220) | 16.345(12.422-21.249) | 2089.048(1565.905-2750.948) | 13.526(10.139-17.812) | -0.172(-0.197--0.149) | -0.02(-0.42,0.38) |
| Jordan | 204.644(164.051-254.502) | 12.529(10.044-15.581) | 370.396(289.681-475.227) | 10.195(7.974-13.081) | -0.186(-0.244--0.122) | -0.72(-0.84,-0.59) |
| Kazakhstan | 1413.516(1231.699-1627.828) | 27.203(23.704-31.327) | 1039.188(891.876-1208.470) | 19.150(16.436-22.270) | -0.296(-0.343--0.250) | -1.52(-1.76,-1.28) |
| Kuwait | 67.029(53.123-85.395) | 12.091(9.583-15.405) | 88.916(68.726-112.924) | 10.517(8.129-13.356) | -0.130(-0.194--0.067) | -0.31(-0.42,-0.20) |
| Kyrgyzstan | 382.860(327.845-449.210) | 22.822(19.543-26.777) | 330.033(272.236-400.486) | 14.511(11.970-17.609) | -0.364(-0.422--0.303) | -1.53(-1.67,-1.40) |
| Lao People's Democratic Republic | 420.950(349.587-509.243) | 22.839(18.967-27.630) | 411.398(340.087-493.938) | 17.915(14.810-21.510) | -0.216(-0.274--0.156) | -0.87(-0.92,-0.82) |
| Lebanon | 128.181(102.295-162.727) | 12.256(9.781-15.559) | 139.133(110.400-177.135) | 10.886(8.638-13.860) | -0.112(-0.175--0.053) | -0.25(-0.32,-0.17) |
| Libya | 165.743(131.339-212.766) | 9.152(7.253-11.749) | 121.073(94.933-155.394) | 8.117(6.364-10.418) | -0.113(-0.169--0.056) | -0.33(-0.40,-0.26) |
| Malaysia | 1479.125(1199.524-1803.574) | 22.503(18.249-27.439) | 1493.602(1195.453-1851.522) | 19.617(15.701-24.318) | -0.128(-0.182--0.068) | -0.47(-0.50,-0.44) |
| Maldives | 25.365(20.910-31.360) | 24.148(19.906-29.855) | 16.209(12.937-19.980) | 16.182(12.915-19.946) | -0.330(-0.397--0.244) | -1.15(-1.34,-0.95) |
| Mauritius | 62.463(50.126-77.635) | 18.925(15.187-23.522) | 42.322(33.958-51.523) | 20.405(16.373-24.842) | 0.078(-0.003-0.170) | 0.23(0.22,0.25) |
| Mongolia | 240.108(211.603-280.868) | 26.678(23.511-31.207) | 209.488(181.886-243.894) | 19.279(16.739-22.445) | -0.277(-0.323--0.225) | -0.65(-0.81,-0.48) |
| Morocco | 1149.360(915.467-1463.598) | 11.745(9.355-14.956) | 1044.635(822.985-1314.705) | 10.668(8.405-13.427) | -0.092(-0.142--0.037) | -0.23(-0.31,-0.15) |
| Myanmar | 3075.917(2536.160-3791.585) | 20.817(17.164-25.660) | 2730.076(2264.425-3336.852) | 17.484(14.502-21.370) | -0.160(-0.216--0.103) | -0.58(-0.70,-0.45) |
| Nepal | 7216.557(6194.161-8478.974) | 85.655(73.520-100.638) | 4880.946(4203.329-5693.907) | 52.897(45.554-61.708) | -0.382(-0.429--0.335) | -1.96(-2.14,-1.78) |
| Oman | 227.222(194.621-266.046) | 27.038(23.159-31.658) | 184.523(153.751-224.011) | 15.088(12.572-18.316) | -0.442(-0.487--0.394) | -1.97(-2.03,-1.90) |
| Pakistan | 36156.620(30701.335-42834.122) | 73.426(62.348-86.987) | 62674.268(53254.743-73483.346) | 73.353(62.328-86.004) | -0.001(-0.051-0.050) | 0.15(0.08,0.22) |
| Palestine | 118.457(95.656-148.116) | 12.234(9.879-15.297) | 201.340(158.029-252.656) | 10.783(8.464-13.531) | -0.119(-0.180--0.047) | -0.32(-0.42,-0.23) |
| Philippines | 8355.886(6957.631-9949.783) | 33.139(27.594-39.461) | 10531.496(8805.801-12479.765) | 30.976(25.901-36.707) | -0.065(-0.098--0.033) | -0.17(-0.29,-0.04) |
| Qatar | 17.238(14.094-21.156) | 13.786(11.271-16.919) | 54.688(43.791-68.633) | 11.073(8.866-13.896) | -0.197(-0.248--0.146) | -0.62(-0.69,-0.56) |
| Republic of Korea | 937.976(704.348-1233.512) | 8.249(6.194-10.848) | 495.793(376.869-653.765) | 8.162(6.204-10.762) | -0.011(-0.083-0.058) | -0.06(-0.14,0.02) |
| Saudi Arabia | 866.866(701.739-1067.376) | 13.228(10.708-16.287) | 771.276(601.668-1000.879) | 10.195(7.953-13.230) | -0.229(-0.290--0.165) | -0.86(-0.90,-0.82) |
| Seychelles | 4.316(3.471-5.473) | 18.192(14.629-23.066) | 4.013(3.199-5.016) | 17.147(13.668-21.431) | -0.057(-0.118-0.001) | -0.12(-0.18,-0.05) |
| Singapore | 29.344(23.771-36.138) | 4.519(3.661-5.565) | 36.837(29.279-46.501) | 4.536(3.605-5.726) | 0.004(-0.059-0.067) | -0.20(-0.93,0.52) |
| Socialist Republic of Viet Nam | 10085.392(8626.835-11991.184) | 38.040(32.539-45.228) | 7800.596(6646.309-9204.188) | 31.501(26.840-37.170) | -0.172(-0.226--0.098) | -0.47(-0.55,-0.39) |
| Sri Lanka | 1354.703(1107.308-1653.873) | 24.484(20.013-29.891) | 1017.557(819.191-1250.462) | 19.936(16.050-24.500) | -0.186(-0.245--0.131) | -0.60(-0.65,-0.55) |
| Sudan | 1112.153(909.194-1370.758) | 12.507(10.224-15.415) | 1726.284(1395.579-2168.214) | 10.406(8.413-13.070) | -0.168(-0.229--0.101) | -0.60(-0.67,-0.53) |
| Syrian Arab Republic | 814.438(659.956-1001.572) | 13.753(11.144-16.912) | 388.741(310.149-487.144) | 10.612(8.466-13.298) | -0.228(-0.288--0.166) | -1.02(-1.11,-0.92) |
| Taiwan (Province of China) | 1080.878(853.630-1363.265) | 19.623(15.498-24.750) | 414.169(331.518-520.955) | 14.055(11.250-17.679) | -0.284(-0.327--0.242) | -1.23(-1.49,-0.98) |
| Tajikistan | 363.253(290.306-453.452) | 15.644(12.502-19.528) | 469.807(368.960-594.195) | 13.107(10.294-16.577) | -0.162(-0.214--0.108) | -0.42(-0.53,-0.30) |
| Thailand | 4612.293(3828.140-5519.540) | 27.359(22.707-32.740) | 1716.881(1341.593-2116.364) | 17.580(13.737-21.670) | -0.357(-0.417--0.292) | -1.76(-1.87,-1.64) |
| Timor-Leste | 94.507(79.708-113.937) | 28.412(23.963-34.253) | 108.810(90.974-130.638) | 20.899(17.474-25.092) | -0.264(-0.317--0.200) | -1.23(-1.32,-1.14) |
| Tunisia | 367.317(291.560-464.857) | 11.828(9.389-14.969) | 293.412(233.402-374.806) | 10.609(8.439-13.552) | -0.103(-0.157--0.049) | -0.16(-0.24,-0.08) |
| Turkey | 3113.907(2503.184-3880.350) | 15.198(12.217-18.939) | 2284.898(1787.432-2923.513) | 12.337(9.651-15.785) | -0.188(-0.243--0.134) | -0.87(-1.00,-0.74) |
| Turkmenistan | 254.032(213.526-305.677) | 16.926(14.227-20.367) | 351.670(312.225-402.200) | 23.075(20.487-26.391) | 0.363(0.244-0.491) | 1.18(0.93,1.43) |
| United Arab Emirates | 76.050(61.786-95.431) | 12.903(10.483-16.191) | 149.999(116.623-187.614) | 11.204(8.711-14.014) | -0.132(-0.188--0.070) | -0.21(-0.29,-0.12) |
| Uzbekistan | 2279.919(2007.258-2598.885) | 26.648(23.461-30.376) | 2251.483(1961.465-2576.492) | 22.312(19.438-25.532) | -0.163(-0.215--0.106) | -0.33(-0.44,-0.22) |
| Yemen | 869.829(704.276-1065.995) | 12.261(9.927-15.026) | 1458.446(1182.115-1812.232) | 10.577(8.573-13.143) | -0.137(-0.197--0.079) | -0.33(-0.48,-0.19) |

**Supplementary table 4:** Mortality rate of encephalitis among males children in Asian countries and regions from 1990 to 2021.

| **level** | **1990** | | **2021** | | **1990–2021** | |
| --- | --- | --- | --- | --- | --- | --- |
|  | **Number (95 %UI)** | **Rate per 100,000(95%UI)** | **Number (95 %UI)** | **Rate per 100,000(95%UI)** | **Rate change** | **Rate EAPC(95 %CI)** |
| Afghanistan | 13.046(5.237-43.159) | 0.594(0.238-1.965) | 37.281(18.393-79.813) | 0.506(0.250-1.084) | -0.148(-0.575-0.979) | -0.16(-0.34,0.02) |
| Algeria | 21.474(11.543-53.514) | 0.391(0.210-0.976) | 19.371(10.425-32.671) | 0.283(0.152-0.478) | -0.276(-0.742-0.509) | -0.36(-0.64,-0.08) |
| Armenia | 2.583(1.738-3.818) | 0.483(0.325-0.713) | 1.433(0.910-2.112) | 0.457(0.290-0.672) | -0.054(-0.516-0.713) | 1.78(0.87,2.70) |
| Azerbaijan | 34.717(12.744-70.694) | 2.785(1.022-5.672) | 27.121(9.259-58.607) | 2.152(0.735-4.650) | -0.227(-0.734-1.399) | -0.36(-0.58,-0.13) |
| Bahrain | 0.158(0.102-0.236) | 0.189(0.122-0.282) | 0.162(0.086-0.245) | 0.106(0.057-0.160) | -0.438(-0.726--0.007) | -0.73(-2.00,0.56) |
| Bangladesh | 5.477(0.707-43.192) | 0.022(0.003-0.172) | 7.104(1.698-33.694) | 0.031(0.007-0.145) | 0.398(-0.592-4.725) | 2.71(-0.35,5.87) |
| Bhutan | 5.965(0.328-12.062) | 4.389(0.241-8.875) | 2.963(0.597-6.000) | 3.142(0.633-6.363) | -0.284(-0.707-2.515) | -1.45(-1.74,-1.16) |
| Brunei Darussalam | 0.079(0.039-0.123) | 0.169(0.082-0.262) | 0.031(0.019-0.055) | 0.063(0.038-0.110) | -0.627(-0.819--0.119) | -0.43(-1.98,1.15) |
| Cambodia | 43.781(19.202-134.254) | 1.864(0.817-5.715) | 49.191(22.843-82.118) | 1.876(0.871-3.132) | 0.007(-0.682-1.439) | 0.03(-0.01,0.07) |
| China | 5737.508(2541.716-7802.240) | 3.450(1.528-4.692) | 900.961(512.183-1570.426) | 0.650(0.370-1.133) | -0.812(-0.906--0.438) | -5.19(-5.50,-4.87) |
| Democratic People's Republic of Korea | 66.737(30.514-117.160) | 2.221(1.016-3.899) | 32.526(13.096-56.638) | 1.332(0.536-2.319) | -0.400(-0.743-0.134) | -1.62(-1.69,-1.56) |
| Egypt | 328.663(129.579-491.270) | 2.877(1.134-4.301) | 188.254(120.273-274.234) | 0.988(0.631-1.440) | -0.657(-0.799--0.173) | -3.41(-3.63,-3.19) |
| Georgia | 7.990(5.664-11.058) | 1.145(0.812-1.585) | 1.905(1.282-2.777) | 0.496(0.334-0.722) | -0.567(-0.744--0.290) | -1.91(-3.09,-0.71) |
| India | 7759.088(4679.949-11684.688) | 4.566(2.754-6.875) | 4968.363(2566.743-7857.535) | 2.592(1.339-4.099) | -0.432(-0.752-0.002) | -2.08(-2.33,-1.82) |
| Indonesia | 804.016(120.170-1834.732) | 2.315(0.346-5.282) | 784.030(122.335-1233.295) | 2.271(0.354-3.572) | -0.019(-0.627-0.689) | -0.02(-0.18,0.14) |
| Iraq | 137.564(62.613-239.022) | 3.250(1.479-5.648) | 137.512(61.806-232.090) | 1.984(0.892-3.348) | -0.390(-0.732-0.262) | -1.22(-1.41,-1.03) |
| Islamic Republic of Iran | 56.744(38.456-103.313) | 0.439(0.297-0.799) | 21.607(11.071-29.277) | 0.209(0.107-0.283) | -0.525(-0.857--0.252) | -0.43(-1.03,0.18) |
| Japan | 21.016(20.024-21.883) | 0.178(0.169-0.185) | 7.607(6.811-8.413) | 0.096(0.086-0.106) | -0.460(-0.520--0.399) | -3.25(-3.93,-2.57) |
| Jordan | 3.633(1.358-5.408) | 0.433(0.162-0.645) | 2.175(1.428-3.139) | 0.116(0.076-0.168) | -0.731(-0.849--0.326) | -4.97(-5.75,-4.18) |
| Kazakhstan | 85.173(67.376-106.161) | 3.235(2.559-4.032) | 51.167(34.738-70.759) | 1.836(1.246-2.539) | -0.432(-0.624--0.150) | -2.52(-3.25,-1.79) |
| Kuwait | 0.631(0.522-0.763) | 0.224(0.185-0.270) | 0.662(0.496-0.854) | 0.152(0.114-0.196) | -0.319(-0.504--0.076) | -0.39(-1.31,0.54) |
| Kyrgyzstan | 40.990(30.675-55.401) | 4.835(3.619-6.535) | 15.488(11.675-19.438) | 1.330(1.003-1.670) | -0.725(-0.816--0.582) | -4.55(-5.02,-4.09) |
| Lao People's Democratic Republic | 16.420(6.654-64.253) | 1.759(0.713-6.882) | 23.122(10.809-40.132) | 1.978(0.924-3.432) | 0.124(-0.700-1.949) | 0.49(0.38,0.60) |
| Lebanon | 1.237(0.615-2.486) | 0.227(0.113-0.457) | 0.922(0.473-1.986) | 0.138(0.071-0.297) | -0.394(-0.747-0.218) | -1.30(-1.66,-0.94) |
| Libya | 2.528(1.229-5.470) | 0.280(0.136-0.607) | 1.647(0.819-3.151) | 0.216(0.107-0.413) | -0.231(-0.659-0.656) | -0.70(-0.79,-0.61) |
| Malaysia | 32.767(18.679-63.937) | 0.972(0.554-1.897) | 26.188(15.222-49.247) | 0.668(0.388-1.256) | -0.313(-0.651-0.228) | -1.02(-1.21,-0.83) |
| Maldives | 0.404(0.145-1.026) | 0.755(0.272-1.919) | 0.201(0.112-0.312) | 0.390(0.217-0.604) | -0.484(-0.846-0.397) | -2.19(-2.53,-1.84) |
| Mauritius | 0.992(0.825-1.197) | 0.593(0.493-0.716) | 1.178(0.916-1.489) | 1.122(0.872-1.417) | 0.890(0.405-1.535) | 3.61(2.75,4.47) |
| Mongolia | 25.807(12.515-49.487) | 5.678(2.754-10.888) | 11.953(6.245-23.585) | 2.148(1.122-4.238) | -0.622(-0.847--0.038) | -2.75(-2.94,-2.56) |
| Morocco | 23.237(10.894-60.537) | 0.470(0.220-1.224) | 14.765(7.272-27.056) | 0.294(0.145-0.539) | -0.374(-0.767-0.498) | -0.84(-1.19,-0.49) |
| Myanmar | 159.836(42.517-401.601) | 2.144(0.570-5.387) | 182.964(51.996-333.868) | 2.303(0.655-4.203) | 0.074(-0.677-1.353) | 0.36(0.06,0.67) |
| Nepal | 217.981(21.882-478.199) | 5.053(0.507-11.085) | 122.637(20.444-226.352) | 2.591(0.432-4.781) | -0.487(-0.824-0.331) | -2.09(-2.20,-1.98) |
| Oman | 13.012(7.294-23.397) | 3.038(1.703-5.463) | 4.811(2.556-11.280) | 0.771(0.410-1.808) | -0.746(-0.918--0.302) | -4.47(-4.86,-4.08) |
| Pakistan | 566.108(111.321-970.873) | 2.213(0.435-3.795) | 1242.274(193.889-2277.398) | 2.804(0.438-5.140) | 0.267(-0.336-1.417) | 1.11(0.99,1.23) |
| Palestine | 0.786(0.410-1.749) | 0.158(0.082-0.351) | 1.941(1.045-3.320) | 0.203(0.109-0.347) | 0.284(-0.655-2.014) | 2.39(1.21,3.58) |
| Philippines | 307.270(99.437-595.841) | 2.379(0.770-4.612) | 378.627(106.643-534.006) | 2.152(0.606-3.035) | -0.095(-0.612-0.287) | -0.02(-0.18,0.15) |
| Qatar | 0.181(0.052-0.313) | 0.280(0.080-0.486) | 0.257(0.078-0.431) | 0.102(0.031-0.171) | -0.636(-0.821--0.237) | -2.41(-3.13,-1.69) |
| Republic of Korea | 13.104(8.269-30.497) | 0.222(0.140-0.516) | 6.708(2.945-10.276) | 0.216(0.095-0.331) | -0.027(-0.823-0.754) | -0.22(-0.85,0.41) |
| Saudi Arabia | 27.549(15.214-55.396) | 0.825(0.455-1.658) | 9.712(3.100-28.075) | 0.245(0.078-0.708) | -0.703(-0.941--0.251) | -4.92(-5.45,-4.38) |
| Seychelles | 0.024(0.014-0.058) | 0.195(0.115-0.479) | 0.043(0.016-0.068) | 0.362(0.137-0.572) | 0.854(-0.514-3.085) | 2.21(0.79,3.64) |
| Singapore | 1.496(1.247-1.785) | 0.444(0.370-0.530) | 1.106(0.872-1.385) | 0.271(0.214-0.340) | -0.390(-0.557--0.188) | -1.56(-2.32,-0.79) |
| Socialist Republic of Viet Nam | 585.702(276.411-1000.407) | 4.316(2.037-7.372) | 454.134(176.238-780.172) | 3.520(1.366-6.048) | -0.184(-0.685-0.747) | -0.20(-0.39,-0.01) |
| Sri Lanka | 31.801(20.203-71.997) | 1.134(0.721-2.568) | 16.498(8.554-41.608) | 0.638(0.331-1.609) | -0.438(-0.718--0.128) | -1.89(-2.29,-1.50) |
| Sudan | 26.122(10.218-103.026) | 0.566(0.221-2.232) | 40.072(18.917-78.869) | 0.468(0.221-0.920) | -0.174(-0.705-1.058) | -0.22(-0.42,-0.02) |
| Syrian Arab Republic | 24.621(13.390-43.996) | 0.812(0.441-1.450) | 12.203(5.756-19.168) | 0.654(0.309-1.028) | -0.194(-0.759-0.513) | -0.94(-1.35,-0.52) |
| Taiwan (Province of China) | 12.077(10.391-14.138) | 0.425(0.365-0.497) | 4.428(3.568-5.395) | 0.290(0.233-0.353) | -0.318(-0.459--0.158) | -0.13(-0.76,0.50) |
| Tajikistan | 1.127(0.287-2.489) | 0.096(0.024-0.211) | 1.498(0.486-2.992) | 0.081(0.026-0.162) | -0.155(-0.670-1.047) | -0.28(-0.51,-0.04) |
| Thailand | 212.933(83.545-351.665) | 2.479(0.972-4.093) | 32.615(20.076-68.013) | 0.650(0.400-1.356) | -0.738(-0.881--0.075) | -5.90(-6.67,-5.12) |
| Timor-Leste | 3.091(1.320-11.273) | 1.785(0.763-6.510) | 5.380(2.237-9.267) | 2.002(0.832-3.449) | 0.122(-0.681-1.702) | 0.24(0.13,0.34) |
| Tunisia | 6.275(3.465-12.735) | 0.395(0.218-0.801) | 3.497(1.690-6.097) | 0.243(0.117-0.423) | -0.385(-0.781-0.371) | -1.01(-1.23,-0.79) |
| Turkey | 66.054(30.186-129.207) | 0.628(0.287-1.227) | 27.039(15.843-41.302) | 0.284(0.167-0.434) | -0.547(-0.831--0.051) | -2.38(-2.94,-1.82) |
| Turkmenistan | 9.639(7.299-12.738) | 1.269(0.961-1.677) | 24.118(16.530-35.378) | 3.077(2.109-4.513) | 1.425(0.467-3.057) | 3.66(2.55,4.78) |
| United Arab Emirates | 0.486(0.259-0.828) | 0.161(0.086-0.273) | 0.361(0.156-0.560) | 0.053(0.023-0.082) | -0.671(-0.847--0.340) | -2.59(-3.03,-2.16) |
| Uzbekistan | 166.217(117.844-223.068) | 3.839(2.722-5.152) | 122.577(88.609-162.837) | 2.343(1.694-3.113) | -0.390(-0.626--0.063) | -1.13(-1.52,-0.74) |
| Yemen | 14.881(6.441-49.703) | 0.403(0.174-1.346) | 28.716(14.798-50.393) | 0.406(0.209-0.712) | 0.007(-0.633-1.242) | 0.40(0.14,0.66) |

**Supplementary table 5:** Mortality rate of encephalitis among females children in Asian countries and regions from 1990 to 2021.

| **level** | **1990** | | **2021** | | **1990–2021** | |
| --- | --- | --- | --- | --- | --- | --- |
|  | **Number (95 %UI)** | **Rate per 100,000(95%UI)** | **Number (95 %UI)** | **Rate per 100,000(95%UI)** | **Rate change** | **Rate EAPC(95 %CI)** |
| Afghanistan | 18.733(6.785-33.637) | 0.887(0.321-1.593) | 48.069(22.423-80.332) | 0.703(0.328-1.175) | -0.208(-0.603-0.671) | -0.77(-0.96,-0.58) |
| Algeria | 21.856(12.022-34.373) | 0.417(0.229-0.656) | 17.442(11.050-26.621) | 0.270(0.171-0.412) | -0.353(-0.669-0.318) | -0.97(-1.21,-0.73) |
| Armenia | 3.054(1.999-4.546) | 0.601(0.394-0.895) | 1.119(0.708-1.659) | 0.402(0.254-0.596) | -0.331(-0.651-0.241) | -0.37(-0.83,0.08) |
| Azerbaijan | 56.896(24.914-109.205) | 4.820(2.110-9.251) | 36.083(14.117-77.919) | 3.279(1.283-7.082) | -0.320(-0.767-0.804) | -0.36(-0.71,0.00) |
| Bahrain | 0.142(0.092-0.193) | 0.179(0.116-0.243) | 0.122(0.083-0.172) | 0.085(0.058-0.119) | -0.526(-0.718--0.257) | -1.67(-2.17,-1.16) |
| Bangladesh | 1.090(0.047-0.261) | 0.005(0.000-0.001) | 2.161(0.895-2.519) | 0.010(0.004-0.011) | 1.099(4.382-40.865) | 7.86(3.41,12.50) |
| Bhutan | 5.060(2.007-9.719) | 4.007(1.589-7.696) | 2.338(1.125-4.516) | 2.518(1.211-4.863) | -0.372(-0.731-1.025) | -1.82(-2.11,-1.52) |
| Brunei Darussalam | 0.044(0.027-0.070) | 0.101(0.061-0.160) | 0.027(0.017-0.041) | 0.059(0.039-0.090) | -0.410(-0.683-0.044) | -1.63(-2.57,-0.68) |
| Cambodia | 58.780(21.370-99.391) | 2.542(0.924-4.299) | 35.576(20.234-59.297) | 1.426(0.811-2.377) | -0.439(-0.756-0.475) | -2.23(-2.42,-2.05) |
| China | 4392.677(3481.678-5488.859) | 2.888(2.289-3.609) | 576.072(448.406-716.718) | 0.476(0.370-0.592) | -0.835(-0.883--0.773) | -5.56(-6.02,-5.11) |
| Democratic People's Republic of Korea | 84.818(30.561-152.667) | 2.881(1.038-5.185) | 37.454(17.268-65.129) | 1.606(0.741-2.793) | -0.442(-0.756-0.345) | -2.18(-2.31,-2.04) |
| Egypt | 296.362(210.164-401.444) | 2.755(1.953-3.731) | 136.551(99.690-192.760) | 0.767(0.560-1.083) | -0.722(-0.824--0.561) | -4.23(-4.48,-3.98) |
| Georgia | 2.907(2.031-4.117) | 0.433(0.303-0.614) | 0.911(0.616-1.311) | 0.259(0.175-0.373) | -0.402(-0.637--0.041) | 0.70(-0.98,2.41) |
| India | 13093.909(8054.633-18072.928) | 8.363(5.144-11.543) | 4653.135(3365.657-6782.822) | 2.664(1.927-3.883) | -0.681(-0.803--0.285) | -3.89(-4.28,-3.51) |
| Indonesia | 951.332(546.065-1278.814) | 2.883(1.655-3.875) | 744.668(517.520-1009.272) | 2.273(1.580-3.081) | -0.211(-0.490-0.312) | -0.65(-0.75,-0.55) |
| Iraq | 159.367(94.027-261.522) | 3.980(2.348-6.531) | 135.877(84.698-198.894) | 2.081(1.297-3.046) | -0.477(-0.705--0.020) | -1.86(-2.02,-1.69) |
| Islamic Republic of Iran | 52.804(39.096-73.346) | 0.424(0.314-0.589) | 18.724(14.069-22.850) | 0.191(0.143-0.233) | -0.550(-0.692--0.362) | -0.19(-0.90,0.53) |
| Japan | 17.870(16.919-18.788) | 0.159(0.150-0.167) | 5.725(5.152-6.285) | 0.076(0.069-0.084) | -0.520(-0.578--0.466) | -3.59(-4.22,-2.95) |
| Jordan | 5.361(3.484-7.901) | 0.675(0.439-0.994) | 2.283(1.620-3.183) | 0.129(0.092-0.180) | -0.808(-0.873--0.665) | -6.62(-7.67,-5.55) |
| Kazakhstan | 60.956(47.374-77.482) | 2.378(1.848-3.023) | 33.697(23.274-46.625) | 1.277(0.882-1.766) | -0.463(-0.646--0.192) | -2.72(-3.56,-1.88) |
| Kuwait | 0.461(0.369-0.583) | 0.169(0.136-0.214) | 0.143(0.113-0.180) | 0.035(0.027-0.044) | -0.795(-0.853--0.714) | -2.54(-5.16,0.15) |
| Kyrgyzstan | 26.772(18.596-38.028) | 3.226(2.241-4.582) | 12.722(9.419-16.802) | 1.146(0.848-1.513) | -0.645(-0.775--0.419) | -3.52(-3.97,-3.06) |
| Lao People's Democratic Republic | 18.490(8.111-32.322) | 2.033(0.892-3.554) | 17.853(9.614-29.348) | 1.584(0.853-2.604) | -0.221(-0.619-1.090) | -0.85(-1.00,-0.69) |
| Lebanon | 0.960(0.574-1.514) | 0.191(0.114-0.301) | 0.670(0.424-1.021) | 0.110(0.070-0.168) | -0.424(-0.679-0.103) | -1.24(-1.72,-0.77) |
| Libya | 2.004(1.262-3.063) | 0.220(0.139-0.337) | 1.226(0.745-1.903) | 0.168(0.102-0.261) | -0.236(-0.590-0.456) | -0.51(-0.67,-0.35) |
| Malaysia | 25.229(14.486-41.754) | 0.788(0.452-1.304) | 17.923(11.645-28.107) | 0.485(0.315-0.761) | -0.384(-0.674-0.182) | -1.32(-1.52,-1.13) |
| Maldives | 0.652(0.248-1.112) | 1.265(0.481-2.157) | 0.184(0.121-0.269) | 0.379(0.249-0.553) | -0.701(-0.852--0.136) | -3.59(-3.85,-3.32) |
| Mauritius | 0.907(0.761-1.077) | 0.557(0.467-0.661) | 0.820(0.630-1.036) | 0.801(0.615-1.012) | 0.438(0.040-0.973) | 0.37(-0.51,1.26) |
| Mongolia | 13.686(5.645-29.366) | 3.072(1.267-6.592) | 8.881(4.609-15.849) | 1.676(0.870-2.990) | -0.455(-0.785-0.589) | -1.08(-1.44,-0.71) |
| Morocco | 22.273(11.499-40.226) | 0.460(0.238-0.831) | 14.964(7.573-24.214) | 0.314(0.159-0.507) | -0.319(-0.650-0.368) | -0.90(-1.13,-0.66) |
| Myanmar | 152.283(60.736-277.423) | 2.080(0.830-3.789) | 107.562(62.657-172.393) | 1.402(0.817-2.247) | -0.326(-0.686-0.767) | -1.31(-1.44,-1.19) |
| Nepal | 273.003(117.404-448.770) | 6.641(2.856-10.916) | 100.956(51.100-175.269) | 2.247(1.137-3.901) | -0.662(-0.841--0.256) | -3.51(-3.68,-3.35) |
| Oman | 11.317(6.264-18.865) | 2.746(1.520-4.578) | 1.851(1.154-2.727) | 0.309(0.193-0.455) | -0.887(-0.945--0.756) | -7.45(-8.10,-6.78) |
| Pakistan | 1291.445(884.200-1822.879) | 5.458(3.737-7.705) | 1725.527(1118.564-2638.874) | 4.195(2.719-6.415) | -0.231(-0.558-0.370) | -0.23(-0.51,0.05) |
| Palestine | 1.089(0.658-1.664) | 0.231(0.140-0.354) | 3.430(2.060-5.169) | 0.377(0.226-0.568) | 0.628(-0.122-1.872) | 4.43(3.29,5.59) |
| Philippines | 333.506(250.809-413.213) | 2.712(2.040-3.360) | 340.077(263.021-424.433) | 2.073(1.603-2.587) | -0.236(-0.422-0.026) | -0.66(-0.88,-0.44) |
| Qatar | 0.229(0.127-0.377) | 0.377(0.210-0.622) | 0.229(0.140-0.395) | 0.094(0.058-0.163) | -0.750(-0.874--0.425) | -5.03(-5.70,-4.35) |
| Republic of Korea | 9.724(7.335-12.702) | 0.178(0.134-0.232) | 5.521(3.996-7.465) | 0.186(0.135-0.252) | 0.046(-0.344-0.645) | -0.14(-0.83,0.55) |
| Saudi Arabia | 29.273(15.397-51.037) | 0.911(0.479-1.589) | 8.878(4.767-17.794) | 0.247(0.133-0.495) | -0.729(-0.881--0.329) | -4.97(-5.40,-4.53) |
| Seychelles | 0.011(0.007-0.015) | 0.093(0.064-0.130) | 0.025(0.017-0.036) | 0.220(0.152-0.310) | 1.372(0.382-3.013) | 2.18(1.48,2.87) |
| Singapore | 1.481(1.261-1.783) | 0.474(0.403-0.570) | 1.156(0.917-1.434) | 0.286(0.227-0.355) | -0.397(-0.566--0.203) | -1.17(-2.52,0.20) |
| Socialist Republic of Viet Nam | 461.243(197.693-775.361) | 3.564(1.527-5.991) | 181.647(106.368-282.627) | 1.531(0.897-2.383) | -0.570(-0.784-0.171) | -2.43(-2.79,-2.06) |
| Sri Lanka | 27.962(19.119-39.749) | 1.024(0.700-1.456) | 11.336(7.261-18.698) | 0.450(0.288-0.743) | -0.560(-0.749--0.198) | -2.96(-3.40,-2.52) |
| Sudan | 52.912(12.530-97.833) | 1.237(0.293-2.288) | 48.403(21.688-84.810) | 0.604(0.270-1.058) | -0.512(-0.767-0.268) | -1.78(-2.05,-1.51) |
| Syrian Arab Republic | 22.317(14.355-33.641) | 0.773(0.497-1.165) | 9.806(6.536-15.034) | 0.545(0.364-0.836) | -0.294(-0.611-0.326) | -1.78(-2.29,-1.27) |
| Taiwan (Province of China) | 9.525(7.949-11.279) | 0.357(0.298-0.423) | 4.967(4.002-6.073) | 0.350(0.282-0.428) | -0.020(-0.254-0.296) | 0.61(0.26,0.96) |
| Tajikistan | 0.533(0.209-1.151) | 0.047(0.018-0.101) | 0.559(0.215-1.125) | 0.032(0.012-0.065) | -0.308(-0.765-1.026) | -0.82(-1.05,-0.59) |
| Thailand | 122.093(70.903-193.242) | 1.477(0.858-2.337) | 27.072(18.606-38.016) | 0.570(0.392-0.800) | -0.614(-0.791--0.275) | -3.96(-4.40,-3.51) |
| Timor-Leste | 7.231(1.512-12.953) | 4.534(0.948-8.122) | 7.344(3.111-12.456) | 2.915(1.234-4.943) | -0.357(-0.690-0.839) | -1.99(-2.26,-1.72) |
| Tunisia | 5.706(3.419-9.392) | 0.377(0.226-0.620) | 2.733(1.625-4.235) | 0.206(0.123-0.319) | -0.452(-0.740-0.030) | -1.30(-1.54,-1.06) |
| Turkey | 67.501(35.830-115.098) | 0.678(0.360-1.155) | 23.346(15.352-33.942) | 0.259(0.170-0.377) | -0.618(-0.793--0.211) | -2.93(-3.30,-2.57) |
| Turkmenistan | 7.548(5.371-10.233) | 1.018(0.725-1.381) | 18.751(12.863-26.927) | 2.533(1.738-3.638) | 1.488(0.535-3.094) | 3.26(2.11,4.42) |
| United Arab Emirates | 0.441(0.256-0.661) | 0.154(0.089-0.231) | 0.387(0.253-0.554) | 0.059(0.039-0.085) | -0.615(-0.781--0.315) | -2.15(-2.62,-1.68) |
| Uzbekistan | 131.225(84.868-202.975) | 3.105(2.008-4.803) | 88.860(59.275-128.239) | 1.829(1.220-2.639) | -0.411(-0.668-0.062) | -1.35(-1.75,-0.95) |
| Yemen | 42.904(7.766-83.607) | 1.261(0.228-2.458) | 59.327(18.188-105.489) | 0.884(0.271-1.572) | -0.299(-0.670-0.660) | -0.85(-1.22,-0.47) |

**Supplementary table 6:** Mortality rate of encephalitis among children in Asian countries and regions from 1990 to 2021.

| **level** | **1990** | | **2021** | | **1990–2021** | |
| --- | --- | --- | --- | --- | --- | --- |
|  | **Number (95 %UI)** | **Rate per 100,000(95%UI)** | **Number (95 %UI)** | **Rate per 100,000(95%UI)** | **Rate change** | **Rate EAPC(95 %CI)** |
| Afghanistan | 31.779(13.873-68.972) | 0.738(0.322-1.601) | 85.350(48.033-142.830) | 0.601(0.338-1.006) | -0.185(-0.523-0.533) | -0.53(-0.68,-0.38) |
| Algeria | 43.330(27.326-78.398) | 0.404(0.255-0.731) | 36.813(24.006-53.822) | 0.277(0.180-0.405) | -0.315(-0.618-0.147) | -0.66(-0.92,-0.41) |
| Armenia | 5.637(3.772-8.326) | 0.540(0.362-0.798) | 2.552(1.619-3.751) | 0.431(0.273-0.633) | -0.203(-0.594-0.466) | 0.80(0.18,1.42) |
| Azerbaijan | 91.613(51.913-155.507) | 3.775(2.139-6.408) | 63.204(29.137-123.033) | 2.677(1.234-5.212) | -0.291(-0.702-0.663) | -0.40(-0.70,-0.10) |
| Bahrain | 0.300(0.227-0.389) | 0.184(0.139-0.238) | 0.284(0.192-0.384) | 0.096(0.065-0.129) | -0.480(-0.642--0.250) | -1.16(-2.07,-0.24) |
| Bangladesh | 6.567(0.821-52.291) | 0.013(0.002-0.107) | 9.265(3.107-41.643) | 0.020(0.007-0.091) | 0.508(-0.506-5.820) | 3.75(0.40,7.22) |
| Bhutan | 11.025(2.542-18.774) | 4.205(0.970-7.160) | 5.301(2.396-8.963) | 2.832(1.280-4.789) | -0.326(-0.652-0.984) | -1.62(-1.91,-1.33) |
| Brunei Darussalam | 0.123(0.076-0.174) | 0.136(0.083-0.192) | 0.058(0.041-0.085) | 0.061(0.043-0.089) | -0.549(-0.732--0.178) | -0.64(-1.82,0.56) |
| Cambodia | 102.562(50.772-191.138) | 2.200(1.089-4.101) | 84.767(52.130-128.207) | 1.657(1.019-2.506) | -0.247(-0.627-0.571) | -1.10(-1.20,-1.01) |
| China | 10130.185(6603.875-12813.692) | 3.182(2.074-4.025) | 1477.033(1091.515-2171.553) | 0.569(0.420-0.836) | -0.821(-0.880--0.661) | -5.33(-5.70,-4.96) |
| Democratic People's Republic of Korea | 151.555(76.481-240.813) | 2.547(1.286-4.048) | 69.980(38.994-109.046) | 1.466(0.817-2.284) | -0.425(-0.687-0.047) | -1.93(-2.01,-1.85) |
| Egypt | 625.025(408.329-835.623) | 2.818(1.841-3.767) | 324.805(240.851-457.213) | 0.881(0.654-1.241) | -0.687(-0.788--0.496) | -3.78(-4.01,-3.55) |
| Georgia | 10.897(7.811-15.002) | 0.796(0.571-1.096) | 2.817(1.919-4.094) | 0.383(0.261-0.556) | -0.519(-0.713--0.230) | -1.00(-2.31,0.33) |
| India | 20852.998(14403.138-27182.568) | 6.386(4.411-8.325) | 9621.498(6653.335-13633.457) | 2.626(1.816-3.721) | -0.589(-0.744--0.269) | -3.10(-3.42,-2.78) |
| Indonesia | 1755.348(901.789-2724.786) | 2.591(1.331-4.023) | 1528.698(720.132-2171.194) | 2.272(1.070-3.227) | -0.123(-0.484-0.297) | -0.35(-0.47,-0.22) |
| Iraq | 296.931(189.910-429.974) | 3.605(2.306-5.220) | 273.388(174.640-394.614) | 2.031(1.297-2.931) | -0.437(-0.671--0.071) | -1.55(-1.72,-1.38) |
| Islamic Republic of Iran | 109.548(83.986-156.423) | 0.432(0.331-0.616) | 40.331(29.481-49.591) | 0.200(0.146-0.246) | -0.537(-0.762--0.359) | -0.31(-0.96,0.34) |
| Japan | 38.887(37.348-40.459) | 0.168(0.162-0.175) | 13.332(12.048-14.565) | 0.086(0.078-0.094) | -0.487(-0.540--0.433) | -3.40(-4.06,-2.74) |
| Jordan | 8.994(5.894-12.094) | 0.551(0.361-0.740) | 4.458(3.345-5.803) | 0.123(0.092-0.160) | -0.777(-0.846--0.631) | -5.88(-6.81,-4.95) |
| Kazakhstan | 146.129(114.745-182.966) | 2.812(2.208-3.521) | 84.865(58.019-116.961) | 1.564(1.069-2.155) | -0.444(-0.635--0.171) | -2.60(-3.37,-1.83) |
| Kuwait | 1.093(0.940-1.289) | 0.197(0.170-0.232) | 0.805(0.624-1.019) | 0.095(0.074-0.120) | -0.517(-0.643--0.359) | -0.97(-2.42,0.50) |
| Kyrgyzstan | 67.762(51.540-88.507) | 4.039(3.072-5.276) | 28.210(21.857-35.720) | 1.240(0.961-1.571) | -0.693(-0.789--0.555) | -4.11(-4.55,-3.67) |
| Lao People's Democratic Republic | 34.910(19.097-87.322) | 1.894(1.036-4.738) | 40.975(26.007-61.716) | 1.784(1.133-2.688) | -0.058(-0.622-0.856) | -0.16(-0.28,-0.03) |
| Lebanon | 2.197(1.375-3.447) | 0.210(0.131-0.330) | 1.592(0.999-2.742) | 0.125(0.078-0.215) | -0.407(-0.661-0.032) | -1.27(-1.67,-0.87) |
| Libya | 4.532(2.861-7.726) | 0.250(0.158-0.427) | 2.873(1.870-4.544) | 0.193(0.125-0.305) | -0.230(-0.569-0.319) | -0.60(-0.72,-0.48) |
| Malaysia | 57.995(38.306-97.566) | 0.882(0.583-1.484) | 44.112(29.849-68.261) | 0.579(0.392-0.897) | -0.343(-0.598-0.069) | -1.15(-1.33,-0.96) |
| Maldives | 1.056(0.559-1.829) | 1.005(0.532-1.741) | 0.385(0.265-0.529) | 0.384(0.265-0.528) | -0.618(-0.806--0.177) | -2.94(-3.14,-2.75) |
| Mauritius | 1.899(1.665-2.171) | 0.575(0.504-0.658) | 1.998(1.620-2.390) | 0.963(0.781-1.152) | 0.674(0.301-1.127) | 1.92(1.43,2.40) |
| Mongolia | 39.493(21.733-67.976) | 4.388(2.415-7.553) | 20.834(12.378-34.237) | 1.917(1.139-3.151) | -0.563(-0.787--0.077) | -2.09(-2.34,-1.84) |
| Morocco | 45.510(24.964-93.406) | 0.465(0.255-0.955) | 29.730(16.210-46.862) | 0.304(0.166-0.479) | -0.347(-0.653-0.224) | -0.87(-1.16,-0.58) |
| Myanmar | 312.120(142.985-555.990) | 2.112(0.968-3.763) | 290.526(136.731-463.120) | 1.861(0.876-2.966) | -0.119(-0.599-0.787) | -0.35(-0.55,-0.14) |
| Nepal | 490.983(232.389-834.243) | 5.828(2.758-9.902) | 223.593(97.767-359.113) | 2.423(1.060-3.892) | -0.584(-0.773--0.213) | -2.80(-2.95,-2.66) |
| Oman | 24.330(15.700-36.537) | 2.895(1.868-4.348) | 6.662(4.286-13.271) | 0.545(0.350-1.085) | -0.812(-0.908--0.586) | -5.54(-6.01,-5.08) |
| Pakistan | 1857.553(1127.823-2504.789) | 3.772(2.290-5.087) | 2967.801(1497.699-4507.545) | 3.473(1.753-5.276) | -0.079(-0.427-0.505) | 0.25(0.04,0.46) |
| Palestine | 1.875(1.237-3.006) | 0.194(0.128-0.310) | 5.371(3.343-7.802) | 0.288(0.179-0.418) | 0.485(-0.235-1.613) | 3.54(2.49,4.60) |
| Philippines | 640.776(430.520-899.829) | 2.541(1.707-3.569) | 718.704(411.639-939.680) | 2.114(1.211-2.764) | -0.168(-0.475-0.113) | -0.34(-0.53,-0.15) |
| Qatar | 0.409(0.225-0.639) | 0.327(0.180-0.511) | 0.485(0.280-0.728) | 0.098(0.057-0.147) | -0.700(-0.830--0.485) | -3.77(-4.15,-3.39) |
| Republic of Korea | 22.828(16.232-43.077) | 0.201(0.143-0.379) | 12.229(8.741-16.490) | 0.201(0.144-0.271) | 0.003(-0.576-0.551) | -0.19(-0.85,0.47) |
| Saudi Arabia | 56.822(35.800-96.614) | 0.867(0.546-1.474) | 18.590(9.997-38.590) | 0.246(0.132-0.510) | -0.717(-0.881--0.419) | -4.94(-5.43,-4.46) |
| Seychelles | 0.034(0.023-0.069) | 0.145(0.099-0.292) | 0.068(0.037-0.098) | 0.292(0.160-0.418) | 1.018(-0.270-2.525) | 2.21(1.01,3.43) |
| Singapore | 2.977(2.607-3.403) | 0.458(0.401-0.524) | 2.261(1.865-2.694) | 0.278(0.230-0.332) | -0.393(-0.522--0.235) | -1.36(-2.22,-0.49) |
| Socialist Republic of Viet Nam | 1046.945(611.341-1588.229) | 3.949(2.306-5.990) | 635.781(321.548-1013.417) | 2.568(1.299-4.093) | -0.350(-0.660-0.305) | -0.97(-1.21,-0.73) |
| Sri Lanka | 59.763(44.421-103.617) | 1.080(0.803-1.873) | 27.834(17.214-52.462) | 0.545(0.337-1.028) | -0.495(-0.685--0.225) | -2.36(-2.77,-1.95) |
| Sudan | 79.034(30.053-157.647) | 0.889(0.338-1.773) | 88.474(52.025-143.005) | 0.533(0.314-0.862) | -0.400(-0.688-0.319) | -1.18(-1.41,-0.95) |
| Syrian Arab Republic | 46.938(31.272-68.532) | 0.793(0.528-1.157) | 22.009(14.164-31.054) | 0.601(0.387-0.848) | -0.242(-0.626-0.237) | -1.33(-1.78,-0.88) |
| Taiwan (Province of China) | 21.602(19.267-24.304) | 0.392(0.350-0.441) | 9.395(7.886-10.982) | 0.319(0.268-0.373) | -0.187(-0.323--0.008) | 0.22(-0.24,0.69) |
| Tajikistan | 1.660(0.697-3.181) | 0.071(0.030-0.137) | 2.057(0.890-3.678) | 0.057(0.025-0.103) | -0.197(-0.634-0.818) | -0.41(-0.64,-0.18) |
| Thailand | 335.026(184.624-477.854) | 1.987(1.095-2.834) | 59.687(42.498-98.800) | 0.611(0.435-1.012) | -0.692(-0.820--0.272) | -5.12(-5.77,-4.48) |
| Timor-Leste | 10.322(3.614-18.617) | 3.103(1.086-5.597) | 12.724(7.144-19.338) | 2.444(1.372-3.714) | -0.212(-0.622-0.718) | -1.20(-1.41,-0.98) |
| Tunisia | 11.981(7.911-19.542) | 0.386(0.255-0.629) | 6.230(3.929-9.496) | 0.225(0.142-0.343) | -0.416(-0.668-0.022) | -1.14(-1.36,-0.91) |
| Turkey | 133.555(82.612-218.882) | 0.652(0.403-1.068) | 50.384(35.219-68.984) | 0.272(0.190-0.372) | -0.583(-0.756--0.292) | -2.65(-3.11,-2.18) |
| Turkmenistan | 17.187(13.082-21.989) | 1.145(0.872-1.465) | 42.870(30.915-57.970) | 2.813(2.029-3.804) | 1.456(0.615-2.788) | 3.48(2.36,4.61) |
| United Arab Emirates | 0.927(0.589-1.323) | 0.157(0.100-0.224) | 0.748(0.477-0.990) | 0.056(0.036-0.074) | -0.645(-0.781--0.428) | -2.37(-2.81,-1.93) |
| Uzbekistan | 297.443(218.028-404.923) | 3.477(2.548-4.733) | 211.437(158.389-281.976) | 2.095(1.570-2.794) | -0.397(-0.608--0.063) | -1.21(-1.60,-0.82) |
| Yemen | 57.786(17.180-103.998) | 0.815(0.242-1.466) | 88.044(41.962-145.049) | 0.639(0.304-1.052) | -0.216(-0.576-0.535) | -0.47(-0.81,-0.12) |

**Supplementary table 7:** DALY rate of encephalitis in male children in Asian countries and regions from 1990 to 2021.

| **Nations** | **1990** | | **2021** | | **1990–2021** | |
| --- | --- | --- | --- | --- | --- | --- |
|  | **Number (95 %UI)** | **Rate per 100,000(95%UI)** | **Number (95 %UI)** | **Rate per 100,000(95%UI)** | **Rate change** | **Rate EAPC(95 %CI)** |
| Afghanistan | 1175.221(501.363-3827.494) | 24.892(12.380-52.842) | 3423.800(1805.316-7131.103) | 19.297(10.395-36.237) | -0.225(-0.646-0.640) | -0.68(-0.76,-0.59) |
| Algeria | 1923.390(1064.839-4712.426) | 16.740(15.875-17.586) | 1738.747(974.146-2865.111) | 9.333(8.381-10.243) | -0.442(-0.498--0.387) | -2.99(-3.60,-2.38) |
| Armenia | 234.737(159.484-341.541) | 15.621(8.851-32.325) | 128.171(83.219-187.054) | 19.350(11.005-32.066) | 0.239(-0.607-1.629) | 2.11(1.03,3.19) |
| Azerbaijan | 3035.905(1122.152-6206.874) | 53.505(22.826-174.258) | 2365.555(819.296-5077.799) | 46.502(24.520-96.855) | -0.131(-0.562-0.922) | -0.16(-0.32,0.00) |
| Bahrain | 14.442(9.742-21.220) | 42.185(20.395-107.548) | 15.068(8.648-21.757) | 26.880(13.970-48.046) | -0.363(-0.752-0.470) | -0.83(-1.17,-0.49) |
| Bangladesh | 2398.462(1222.153-6046.565) | 19.914(13.011-44.414) | 2165.804(1121.386-4512.008) | 19.269(8.748-29.069) | -0.032(-0.810-0.684) | -0.23(-0.83,0.38) |
| Bhutan | 530.848(42.018-1050.690) | 165.518(74.299-501.097) | 261.387(58.943-530.407) | 164.147(77.099-273.352) | -0.008(-0.682-1.376) | -0.02(-0.06,0.02) |
| Brunei Darussalam | 7.107(3.624-10.796) | 335.978(239.018-449.958) | 3.067(2.018-5.115) | 205.426(149.136-271.933) | -0.389(-0.625--0.062) | -1.12(-1.50,-0.74) |
| Cambodia | 3888.024(1745.274-11770.752) | 35.064(19.412-85.909) | 4303.252(2021.214-7166.155) | 25.425(14.245-41.896) | -0.275(-0.734-0.468) | -0.37(-0.65,-0.10) |
| China | 517178.511(238248.888-699245.089) | 243.571(90.030-497.978) | 85736.341(51331.467-144271.744) | 187.694(65.007-402.895) | -0.229(-0.733-1.383) | -0.34(-0.57,-0.11) |
| Democratic People's Republic of Korea | 6051.949(2905.886-10488.432) | 201.415(96.711-349.066) | 3000.384(1304.654-5123.899) | 122.854(53.421-209.804) | -0.390(-0.724-0.132) | -1.56(-1.62,-1.49) |
| Egypt | 28687.734(11275.506-42864.404) | 251.140(98.709-375.246) | 16416.607(10579.190-23644.784) | 86.181(55.537-124.126) | -0.657(-0.798--0.181) | -3.40(-3.62,-3.18) |
| Georgia | 708.474(505.968-977.010) | 101.530(72.510-140.014) | 170.931(116.702-248.441) | 44.471(30.362-64.636) | -0.562(-0.738--0.283) | -1.91(-3.04,-0.76) |
| India | 697092.258(431757.541-1031899.120) | 410.174(254.049-607.177) | 448515.194(242784.015-700686.149) | 233.949(126.638-365.483) | -0.430(-0.746--0.017) | -2.09(-2.34,-1.85) |
| Indonesia | 71447.585(11122.338-162647.065) | 205.694(32.021-468.253) | 69050.812(10766.823-108830.985) | 199.978(31.182-315.185) | -0.028(-0.629-0.625) | -0.06(-0.22,0.10) |
| Iraq | 11950.087(5437.514-20737.316) | 282.352(128.476-489.974) | 11972.487(5412.701-20124.878) | 172.719(78.085-290.328) | -0.388(-0.730-0.271) | -1.22(-1.41,-1.03) |
| Islamic Republic of Iran | 5081.884(3493.571-9130.647) | 39.285(27.007-70.583) | 1934.477(1047.242-2591.058) | 18.670(10.107-25.007) | -0.525(-0.845--0.266) | -0.46(-1.05,0.14) |
| Japan | 1981.888(1879.441-2082.014) | 18.163(15.809-26.246) | 740.421(664.865-812.657) | 12.490(9.808-15.113) | -0.312(-0.623--0.164) | -1.87(-2.44,-1.30) |
| Jordan | 327.456(129.470-484.287) | 39.032(15.432-57.725) | 211.046(146.534-288.796) | 11.304(7.849-15.469) | -0.710(-0.831--0.317) | -4.72(-5.45,-3.98) |
| Kazakhstan | 7457.088(5910.366-9282.569) | 283.207(224.465-352.535) | 4499.771(3066.846-6230.406) | 161.459(110.043-223.557) | -0.430(-0.622--0.152) | -2.49(-3.21,-1.76) |
| Kuwait | 57.175(47.609-68.737) | 20.259(16.869-24.355) | 60.606(46.314-77.337) | 13.948(10.659-17.798) | -0.312(-0.489--0.081) | -0.39(-1.27,0.51) |
| Kyrgyzstan | 3607.474(2705.126-4888.657) | 425.550(319.106-576.682) | 1360.760(1026.536-1715.029) | 116.898(88.186-147.332) | -0.725(-0.816--0.582) | -4.55(-5.01,-4.08) |
| Lao People's Democratic Republic | 1449.648(600.714-5625.958) | 155.268(64.341-602.583) | 2015.773(951.839-3472.419) | 172.400(81.407-296.981) | 0.110(-0.700-1.870) | 0.45(0.34,0.56) |
| Lebanon | 114.932(61.206-226.080) | 21.140(11.258-41.585) | 88.150(49.627-181.464) | 13.175(7.417-27.121) | -0.377(-0.719-0.190) | -1.23(-1.55,-0.91) |
| Libya | 224.344(111.579-476.247) | 18.878(11.853-42.806) | 147.365(79.386-276.734) | 33.900(13.751-52.490) | 0.796(-0.468-2.652) | 2.08(0.77,3.40) |
| Malaysia | 2862.722(1675.022-5548.190) | 84.951(49.706-164.642) | 2276.820(1353.267-4261.514) | 58.061(34.509-108.672) | -0.317(-0.650-0.202) | -1.04(-1.22,-0.86) |
| Maldives | 36.223(13.909-89.986) | 67.736(26.010-168.273) | 18.077(10.528-27.588) | 35.033(20.403-53.465) | -0.483(-0.840-0.324) | -2.16(-2.48,-1.84) |
| Mauritius | 89.569(74.873-107.335) | 53.584(44.792-64.212) | 102.795(80.067-129.706) | 97.861(76.225-123.481) | 0.826(0.382-1.424) | 3.43(2.61,4.26) |
| Mongolia | 2286.869(1113.906-4350.689) | 503.167(245.086-957.257) | 1062.096(558.709-2080.949) | 190.827(100.383-373.884) | -0.621(-0.845--0.036) | -2.73(-2.92,-2.54) |
| Morocco | 2086.601(1008.805-5319.674) | 37.916(31.836-44.996) | 1349.296(701.227-2411.751) | 23.021(18.159-28.566) | -0.393(-0.557--0.192) | -1.57(-2.33,-0.80) |
| Myanmar | 14301.379(3944.151-35491.149) | 191.830(52.904-476.056) | 16217.492(4614.793-29587.925) | 204.175(58.099-372.506) | 0.064(-0.678-1.327) | 0.33(0.02,0.63) |
| Nepal | 19338.776(2450.025-42231.133) | 448.278(56.792-978.929) | 10881.614(2145.068-19843.039) | 229.862(45.312-419.162) | -0.487(-0.820-0.238) | -2.11(-2.21,-2.00) |
| Oman | 1137.218(639.500-2048.721) | 265.548(149.328-478.390) | 422.942(229.037-978.350) | 67.796(36.714-156.827) | -0.745(-0.916--0.308) | -4.43(-4.80,-4.06) |
| Pakistan | 50371.642(12494.156-84591.348) | 196.902(48.839-330.666) | 110097.484(22032.620-197221.821) | 248.481(49.726-445.114) | 0.262(-0.313-1.329) | 1.10(0.98,1.22) |
| Palestine | 77.758(44.060-160.906) | 17.236(11.627-25.324) | 185.209(105.336-306.916) | 9.881(5.671-14.268) | -0.427(-0.693--0.029) | -0.73(-1.93,0.48) |
| Philippines | 27073.065(9086.777-52009.388) | 209.574(70.341-402.607) | 33203.209(9741.992-46759.070) | 188.721(55.372-265.770) | -0.100(-0.608-0.277) | -0.04(-0.20,0.12) |
| Qatar | 16.187(5.014-27.476) | 25.114(7.779-42.628) | 23.475(8.243-38.266) | 9.326(3.275-15.202) | -0.629(-0.806--0.247) | -2.40(-3.08,-1.71) |
| Republic of Korea | 1175.876(768.234-2622.492) | 50.565(20.902-196.208) | 598.858(271.889-903.438) | 41.673(20.724-81.339) | -0.176(-0.704-1.002) | -0.25(-0.44,-0.06) |
| Saudi Arabia | 2409.575(1345.370-4831.503) | 72.117(40.266-144.604) | 854.061(295.386-2431.246) | 21.523(7.444-61.270) | -0.702(-0.934--0.258) | -4.88(-5.41,-4.35) |
| Seychelles | 2.275(1.429-5.159) | 217.443(86.498-356.876) | 4.032(1.636-6.244) | 57.035(35.858-117.301) | -0.738(-0.878--0.104) | -5.89(-6.65,-5.12) |
| Singapore | 127.651(107.180-151.486) | 38.512(33.578-44.602) | 93.845(74.023-116.448) | 25.835(20.993-31.327) | -0.329(-0.463--0.176) | -0.24(-0.83,0.35) |
| Socialist Republic of Viet Nam | 52135.304(25061.389-88947.842) | 384.189(184.679-655.463) | 39979.914(15864.567-68311.119) | 309.918(122.980-529.538) | -0.193(-0.684-0.718) | -0.23(-0.42,-0.04) |
| Sri Lanka | 2845.003(1847.571-6240.562) | 113.320(85.961-149.347) | 1482.779(792.491-3630.761) | 270.998(185.763-397.404) | 1.391(0.450-2.994) | 3.61(2.52,4.71) |
| Sudan | 2334.372(964.970-9058.027) | 390.597(30.917-773.096) | 3571.673(1776.243-6971.373) | 277.200(62.509-562.496) | -0.290(-0.696-1.713) | -1.47(-1.75,-1.19) |
| Syrian Arab Republic | 2160.149(1208.963-3859.037) | 71.217(39.858-127.226) | 1050.445(516.351-1625.400) | 56.315(27.682-87.139) | -0.209(-0.749-0.461) | -0.99(-1.38,-0.60) |
| Taiwan (Province of China) | 1094.913(954.631-1268.045) | 259.075(213.773-316.900) | 394.911(320.891-478.855) | 157.806(123.095-204.723) | -0.391(-0.569--0.173) | -1.50(-1.83,-1.16) |
| Tajikistan | 116.462(42.502-236.134) | 9.891(3.610-20.055) | 159.472(73.079-280.646) | 8.615(3.948-15.161) | -0.129(-0.605-0.885) | -0.38(-0.58,-0.18) |
| Thailand | 18680.942(7431.210-30659.837) | 43.852(29.794-63.804) | 2859.970(1798.071-5881.947) | 40.817(26.502-59.569) | -0.069(-0.509-0.643) | 1.65(0.76,2.53) |
| Timor-Leste | 273.739(119.807-993.229) | 158.091(69.191-573.612) | 470.809(197.722-812.005) | 175.241(73.595-302.239) | 0.108(-0.680-1.650) | 0.19(0.08,0.30) |
| Tunisia | 566.173(326.047-1131.309) | 35.607(20.505-71.148) | 317.915(166.051-537.995) | 22.075(11.530-37.356) | -0.380(-0.768-0.321) | -1.01(-1.22,-0.79) |
| Turkey | 5817.782(2724.051-11328.640) | 55.270(25.879-107.623) | 2396.583(1453.533-3576.668) | 25.210(15.290-37.624) | -0.544(-0.825--0.070) | -2.37(-2.91,-1.83) |
| Turkmenistan | 860.793(652.967-1134.457) | 15.163(7.732-23.032) | 2124.226(1456.109-3115.066) | 6.217(4.092-10.369) | -0.590(-0.785--0.106) | -0.31(-1.78,1.18) |
| United Arab Emirates | 43.758(24.311-72.607) | 14.448(8.027-23.973) | 36.791(19.269-54.242) | 5.367(2.811-7.913) | -0.629(-0.812--0.288) | -2.31(-2.70,-1.91) |
| Uzbekistan | 14547.399(10349.167-19482.534) | 310.988(143.263-420.468) | 10747.186(7802.278-14226.601) | 61.874(37.045-104.118) | -0.801(-0.892--0.436) | -5.02(-5.32,-4.72) |
| Yemen | 1343.444(607.704-4402.319) | 36.380(16.456-119.214) | 2601.526(1436.904-4489.352) | 36.747(20.297-63.413) | 0.010(-0.622-1.152) | 0.40(0.15,0.65) |

**Supplementary table 8:** DALY rate of encephalitis among female children in Asian countries and regions from 1990 to 2021.

| **Nations** | **1990** | | **2021** | | **1990–2021** | |
| --- | --- | --- | --- | --- | --- | --- |
|  | **Number (95 %UI)** | **Rate per 100,000(95%UI)** | **Number (95 %UI)** | **Rate per 100,000(95%UI)** | **Rate change** | **Rate EAPC(95 %CI)** |
| Afghanistan | 1694.562(643.567-2998.745) | 19.881(12.754-29.911) | 4400.464(2179.468-7276.191) | 15.428(9.794-23.106) | -0.224(-0.568-0.430) | -0.48(-0.63,-0.33) |
| Algeria | 1984.563(1123.408-3143.936) | 15.054(14.273-15.873) | 1585.734(1044.504-2390.768) | 7.565(6.851-8.243) | -0.497(-0.549--0.446) | -3.28(-3.85,-2.72) |
| Armenia | 274.700(182.675-404.100) | 22.155(14.201-33.359) | 100.444(64.859-147.565) | 34.415(21.538-51.459) | 0.553(-0.117-1.661) | 4.03(2.99,5.07) |
| Azerbaijan | 5054.241(2208.995-9681.810) | 80.250(30.477-142.012) | 3203.952(1253.422-6939.238) | 64.354(31.873-106.409) | -0.198(-0.590-0.626) | -0.76(-0.95,-0.56) |
| Bahrain | 13.308(8.896-17.852) | 41.617(22.365-73.676) | 11.858(8.244-16.210) | 28.520(15.358-45.253) | -0.315(-0.633-0.328) | -0.89(-1.12,-0.66) |
| Bangladesh | 1823.065(992.240-3097.053) | 16.101(12.268-20.851) | 1505.615(778.679-2682.969) | 16.601(12.150-22.229) | 0.031(-0.336-0.590) | -0.16(-0.83,0.51) |
| Bhutan | 447.636(187.024-852.556) | 224.997(82.993-380.212) | 203.540(99.898-389.802) | 124.416(71.106-206.229) | -0.447(-0.755-0.443) | -2.28(-2.47,-2.10) |
| Brunei Darussalam | 4.050(2.535-6.370) | 272.139(175.748-423.145) | 2.599(1.759-3.809) | 160.284(107.268-231.334) | -0.411(-0.670-0.070) | -1.35(-1.74,-0.96) |
| Cambodia | 5201.806(1918.761-8790.289) | 37.879(21.442-60.007) | 3104.156(1774.082-5145.368) | 24.535(16.161-36.991) | -0.352(-0.657-0.281) | -0.99(-1.22,-0.75) |
| China | 397049.209(315501.030-492605.463) | 428.136(187.120-820.129) | 55359.429(43859.622-68132.075) | 291.200(113.921-630.692) | -0.320(-0.766-0.812) | -0.35(-0.71,0.01) |
| Democratic People's Republic of Korea | 7645.458(2838.657-13628.842) | 259.648(96.404-462.850) | 3416.314(1620.550-5870.877) | 146.530(69.507-251.809) | -0.436(-0.745-0.314) | -2.13(-2.26,-2.00) |
| Egypt | 25896.072(18350.829-35140.017) | 240.698(170.567-326.619) | 12005.433(8812.413-16784.665) | 67.423(49.491-94.263) | -0.720(-0.822--0.562) | -4.20(-4.45,-3.95) |
| Georgia | 264.756(187.823-369.577) | 39.462(27.995-55.086) | 83.735(58.808-119.779) | 23.817(16.727-34.070) | -0.396(-0.626--0.046) | 0.57(-1.02,2.19) |
| India | 1170894.304(725711.210-1609005.569) | 747.838(463.504-1027.655) | 417569.451(303638.558-600276.042) | 239.043(173.822-343.635) | -0.680(-0.799--0.294) | -3.91(-4.28,-3.53) |
| Indonesia | 84056.517(48436.719-112735.210) | 254.698(146.767-341.597) | 65078.813(45252.043-88155.149) | 198.658(138.135-269.101) | -0.220(-0.496-0.296) | -0.70(-0.79,-0.60) |
| Iraq | 13896.806(8283.683-22656.557) | 347.061(206.878-565.829) | 11822.558(7440.036-17350.692) | 181.046(113.934-265.702) | -0.478(-0.706--0.031) | -1.88(-2.05,-1.71) |
| Islamic Republic of Iran | 4795.205(3604.196-6612.743) | 38.520(28.953-53.121) | 1711.033(1300.053-2085.909) | 17.427(13.241-21.245) | -0.548(-0.684--0.367) | -0.25(-0.93,0.44) |
| Japan | 1693.623(1605.756-1785.716) | 15.851(14.408-17.518) | 568.208(514.608-619.131) | 10.646(9.394-12.251) | -0.328(-0.433--0.209) | -1.97(-2.53,-1.42) |
| Jordan | 475.519(315.272-689.837) | 59.856(39.685-86.833) | 221.521(164.497-299.874) | 12.543(9.314-16.980) | -0.790(-0.859--0.656) | -6.28(-7.28,-5.28) |
| Kazakhstan | 5340.652(4170.859-6765.558) | 208.363(162.724-263.956) | 2959.386(2057.101-4104.903) | 112.118(77.934-155.516) | -0.462(-0.646--0.190) | -2.68(-3.50,-1.84) |
| Kuwait | 42.903(34.734-53.558) | 15.766(12.764-19.682) | 15.932(13.088-19.675) | 3.877(3.185-4.788) | -0.754(-0.819--0.673) | -2.24(-4.62,0.21) |
| Kyrgyzstan | 2363.529(1636.438-3370.543) | 284.812(197.195-406.160) | 1123.173(832.223-1481.124) | 101.157(74.953-133.395) | -0.645(-0.776--0.428) | -3.51(-3.97,-3.05) |
| Lao People's Democratic Republic | 1639.150(717.813-2842.012) | 180.231(78.927-312.491) | 1552.076(838.752-2546.154) | 137.702(74.415-225.898) | -0.236(-0.623-1.033) | -0.92(-1.06,-0.77) |
| Lebanon | 91.363(57.397-137.887) | 18.192(11.429-27.456) | 66.356(45.442-97.142) | 10.896(7.462-15.952) | -0.401(-0.646-0.052) | -1.18(-1.59,-0.77) |
| Libya | 180.848(116.016-272.091) | 9.644(6.906-12.907) | 112.308(71.296-168.201) | 20.158(14.417-27.669) | 1.090(0.324-2.296) | 1.86(1.25,2.48) |
| Malaysia | 2212.286(1303.479-3645.357) | 69.066(40.694-113.805) | 1562.174(1029.207-2406.688) | 42.310(27.875-65.183) | -0.387(-0.670-0.142) | -1.34(-1.53,-1.16) |
| Maldives | 58.053(22.452-97.912) | 112.583(43.542-189.881) | 16.558(11.160-24.158) | 34.091(22.977-49.739) | -0.697(-0.846--0.140) | -3.55(-3.81,-3.29) |
| Mauritius | 80.332(67.777-94.559) | 49.314(41.607-58.047) | 69.514(54.701-86.767) | 67.909(53.438-84.764) | 0.377(0.017-0.852) | 0.32(-0.53,1.18) |
| Mongolia | 1211.822(493.568-2594.490) | 272.007(110.787-582.363) | 790.422(413.652-1409.835) | 149.128(78.043-265.992) | -0.452(-0.785-0.597) | -1.06(-1.43,-0.69) |
| Morocco | 2014.017(1082.325-3565.490) | 41.073(35.019-49.273) | 1361.037(732.933-2159.573) | 24.726(19.624-30.676) | -0.398(-0.567--0.201) | -1.18(-2.53,0.18) |
| Myanmar | 13615.103(5512.062-24573.701) | 185.971(75.290-335.656) | 9499.067(5491.661-15210.295) | 123.818(71.582-198.262) | -0.334(-0.689-0.722) | -1.36(-1.49,-1.23) |
| Nepal | 23974.655(10851.426-38969.341) | 583.159(263.950-947.890) | 8852.905(4732.710-15145.842) | 197.027(105.329-337.080) | -0.662(-0.836--0.273) | -3.54(-3.71,-3.38) |
| Oman | 997.626(551.568-1649.516) | 242.071(133.837-400.251) | 166.405(106.697-244.203) | 27.773(17.808-40.757) | -0.885(-0.944--0.751) | -7.36(-7.98,-6.74) |
| Pakistan | 113914.548(78394.401-159931.121) | 481.467(331.339-675.959) | 149392.916(97681.864-228134.993) | 363.187(237.473-554.616) | -0.246(-0.565-0.344) | -0.27(-0.56,0.02) |
| Palestine | 104.239(66.815-156.956) | 16.749(11.196-22.468) | 313.186(196.000-468.291) | 8.222(5.716-11.239) | -0.509(-0.692--0.259) | -1.61(-2.08,-1.14) |
| Philippines | 29330.210(22157.206-36292.846) | 238.531(180.196-295.155) | 29699.730(22956.559-36881.034) | 181.044(139.939-224.820) | -0.241(-0.426-0.016) | -0.69(-0.91,-0.47) |
| Qatar | 20.494(11.616-33.444) | 33.824(19.172-55.198) | 21.338(13.820-35.341) | 8.810(5.706-14.592) | -0.740(-0.862--0.427) | -4.91(-5.54,-4.27) |
| Republic of Korea | 880.053(670.572-1139.673) | 110.769(27.266-202.822) | 492.494(360.453-659.453) | 54.221(25.137-93.970) | -0.511(-0.762-0.241) | -1.79(-2.05,-1.52) |
| Saudi Arabia | 2571.575(1377.692-4457.360) | 80.057(42.890-138.764) | 780.556(441.978-1539.473) | 21.699(12.287-42.797) | -0.729(-0.880--0.347) | -4.94(-5.37,-4.51) |
| Seychelles | 1.126(0.806-1.507) | 129.911(76.075-204.974) | 2.320(1.660-3.185) | 49.956(34.790-69.618) | -0.615(-0.790--0.298) | -3.96(-4.40,-3.51) |
| Singapore | 128.438(109.506-154.081) | 32.703(27.745-38.210) | 100.005(79.369-124.066) | 30.997(25.208-37.707) | -0.052(-0.267-0.223) | 0.46(0.14,0.79) |
| Socialist Republic of Viet Nam | 41180.791(17930.133-68725.771) | 318.189(138.540-531.019) | 15999.242(9488.902-24802.586) | 134.873(79.991-209.084) | -0.576(-0.787-0.120) | -2.48(-2.83,-2.12) |
| Sri Lanka | 2499.556(1740.963-3498.614) | 91.162(65.155-123.264) | 1013.608(671.251-1630.650) | 223.884(154.158-321.232) | 1.456(0.518-3.036) | 3.21(2.08,4.35) |
| Sudan | 4736.359(1165.843-8672.441) | 354.457(148.093-675.089) | 4347.689(2015.589-7534.993) | 219.164(107.567-419.725) | -0.382(-0.728-0.891) | -1.86(-2.13,-1.58) |
| Syrian Arab Republic | 1973.854(1287.745-2951.750) | 68.326(44.576-102.176) | 853.843(581.995-1280.688) | 47.488(32.368-71.227) | -0.305(-0.607-0.288) | -1.80(-2.29,-1.32) |
| Taiwan (Province of China) | 871.570(739.442-1018.335) | 217.316(167.567-275.288) | 439.583(357.490-534.737) | 133.294(100.397-171.867) | -0.387(-0.575--0.099) | -1.39(-1.78,-1.00) |
| Tajikistan | 65.070(33.847-120.007) | 5.685(2.957-10.485) | 78.252(44.782-130.384) | 4.515(2.584-7.523) | -0.206(-0.638-0.608) | -0.77(-1.04,-0.51) |
| Thailand | 10740.268(6289.443-16946.039) | 54.084(35.966-79.561) | 2373.870(1653.173-3308.172) | 36.085(23.301-53.013) | -0.333(-0.641-0.215) | -0.43(-0.86,0.01) |
| Timor-Leste | 639.024(134.364-1136.963) | 400.699(84.253-712.931) | 642.634(272.021-1086.622) | 255.037(107.955-431.239) | -0.364(-0.693-0.788) | -2.03(-2.30,-1.75) |
| Tunisia | 522.812(315.682-847.147) | 34.500(20.831-55.902) | 254.343(161.786-388.401) | 19.188(12.205-29.301) | -0.444(-0.721-0.014) | -1.28(-1.51,-1.05) |
| Turkey | 6030.149(3288.742-10186.757) | 60.528(33.011-102.251) | 2100.140(1412.776-3000.745) | 23.299(15.673-33.290) | -0.615(-0.784--0.231) | -2.93(-3.28,-2.58) |
| Turkmenistan | 675.740(482.960-913.694) | 9.265(5.800-14.574) | 1657.079(1140.996-2377.596) | 5.741(3.885-8.414) | -0.380(-0.653-0.053) | -1.48(-2.34,-0.62) |
| United Arab Emirates | 40.067(24.476-58.867) | 13.984(8.542-20.545) | 38.713(26.971-52.836) | 5.925(4.128-8.087) | -0.576(-0.746--0.277) | -1.95(-2.37,-1.52) |
| Uzbekistan | 11499.989(7426.720-17881.151) | 261.088(207.464-323.923) | 7788.855(5212.585-11241.482) | 45.727(36.228-56.277) | -0.825(-0.873--0.763) | -5.37(-5.80,-4.95) |
| Yemen | 3850.842(728.373-7475.625) | 113.208(21.413-219.770) | 5332.657(1690.353-9404.680) | 79.485(25.195-140.179) | -0.298(-0.666-0.646) | -0.85(-1.22,-0.48) |

**Supplementary table 9:** DALY rate of encephalitis among children in Asian countries from 1990 to 2021.

| **level** | **1990** | | **2021** | | **1990–2021** | |
| --- | --- | --- | --- | --- | --- | --- |
|  | **Number (95 %UI)** | **Rate per 100,000(95%UI)** | **Number (95 %UI)** | **Rate per 100,000(95%UI)** | **Rate change** | **Rate EAPC(95 %CI)** |
| Afghanistan | 2869.783(1308.367-6137.982) | 66.614(30.370-142.476) | 7824.264(4646.148-12974.077) | 55.098(32.718-91.363) | -0.173(-0.510-0.506) | -0.52(-0.66,-0.37) |
| Algeria | 3907.953(2496.663-6963.730) | 36.439(23.280-64.932) | 3324.480(2211.451-4782.267) | 24.993(16.625-35.952) | -0.314(-0.609-0.129) | -0.68(-0.93,-0.43) |
| Armenia | 509.437(342.866-745.943) | 8.631(5.019-17.307) | 228.615(148.887-333.943) | 8.022(4.815-14.331) | -0.071(-0.407-0.434) | 0.72(-0.72,2.18) |
| Azerbaijan | 8090.146(4604.781-13651.644) | 333.348(189.736-562.505) | 5569.507(2581.341-10786.605) | 235.938(109.352-456.946) | -0.292(-0.705-0.659) | -0.39(-0.69,-0.09) |
| Bahrain | 27.750(21.400-35.317) | 79.514(30.857-157.157) | 26.926(19.223-35.373) | 47.738(28.620-76.427) | -0.400(-0.689-0.289) | -1.20(-1.42,-0.97) |
| Bangladesh | 4221.527(2454.931-8465.342) | 18.796(13.061-28.646) | 3671.419(2203.855-6558.905) | 26.693(17.641-37.622) | 0.420(-0.233-1.387) | 3.18(2.22,4.14) |
| Bhutan | 978.484(248.539-1655.590) | 39.436(34.634-44.982) | 464.927(216.836-783.799) | 23.870(19.696-28.341) | -0.395(-0.523--0.236) | -1.37(-2.23,-0.50) |
| Brunei Darussalam | 11.157(7.091-15.560) | 12.317(7.829-17.179) | 5.665(4.134-7.980) | 5.989(4.370-8.435) | -0.514(-0.698--0.154) | -0.54(-1.65,0.58) |
| Cambodia | 9089.830(4546.962-16850.344) | 35.701(31.974-39.907) | 7407.407(4559.994-11096.342) | 28.319(24.036-33.090) | -0.207(-0.334--0.039) | 0.09(-0.34,0.53) |
| China | 914227.720(602368.475-1150465.144) | 287.153(189.200-361.354) | 141095.770(107721.420-202509.447) | 54.345(41.490-77.999) | -0.811(-0.870--0.653) | -5.15(-5.50,-4.80) |
| Democratic People's Republic of Korea | 13697.407(7120.846-21551.532) | 230.237(119.693-362.256) | 6416.698(3674.124-9889.529) | 134.417(76.966-207.166) | -0.416(-0.679-0.040) | -1.87(-1.94,-1.80) |
| Egypt | 54583.806(35594.663-72963.855) | 246.075(160.468-328.937) | 28422.041(21265.238-39962.798) | 77.118(57.699-108.432) | -0.687(-0.787--0.496) | -3.76(-3.99,-3.53) |
| Georgia | 973.230(703.575-1337.451) | 71.106(51.404-97.716) | 254.666(176.833-369.468) | 34.604(24.028-50.204) | -0.513(-0.706--0.226) | -1.03(-2.28,0.24) |
| India | 1867986.562(1300842.296-2409788.787) | 572.088(398.395-738.020) | 866084.645(607994.952-1216861.727) | 236.377(165.938-332.114) | -0.587(-0.739--0.279) | -3.12(-3.43,-2.80) |
| Indonesia | 155504.102(79797.961-241475.076) | 229.570(117.805-356.488) | 134129.625(62932.180-190647.308) | 199.336(93.526-283.329) | -0.132(-0.490-0.280) | -0.39(-0.51,-0.27) |
| Iraq | 25846.893(16526.315-37374.488) | 313.811(200.648-453.769) | 23795.045(15217.824-34193.896) | 176.758(113.044-254.005) | -0.437(-0.672--0.067) | -1.56(-1.74,-1.39) |
| Islamic Republic of Iran | 9877.088(7627.907-13967.072) | 38.910(30.049-55.022) | 3645.509(2731.375-4437.345) | 18.065(13.535-21.989) | -0.536(-0.751--0.366) | -0.36(-0.99,0.28) |
| Japan | 3675.511(3499.606-3849.357) | 15.919(15.157-16.672) | 1308.629(1189.304-1425.697) | 8.473(7.701-9.231) | -0.468(-0.517--0.419) | -3.12(-3.71,-2.53) |
| Jordan | 802.975(530.855-1072.240) | 49.160(32.500-65.645) | 432.566(340.609-550.933) | 11.906(9.375-15.165) | -0.758(-0.828--0.618) | -5.59(-6.46,-4.71) |
| Kazakhstan | 12797.740(10073.538-16056.171) | 246.289(193.862-308.996) | 7459.157(5138.138-10276.799) | 137.458(94.686-189.382) | -0.442(-0.631--0.168) | -2.56(-3.32,-1.79) |
| Kuwait | 100.079(86.561-116.697) | 18.053(15.615-21.051) | 76.538(61.198-94.747) | 9.052(7.238-11.206) | -0.499(-0.619--0.346) | -0.93(-2.30,0.46) |
| Kyrgyzstan | 5971.003(4521.735-7812.715) | 355.930(269.540-465.714) | 2483.933(1940.077-3143.743) | 109.213(85.301-138.224) | -0.693(-0.789--0.555) | -4.11(-4.54,-3.66) |
| Lao People's Democratic Republic | 3088.798(1693.730-7675.021) | 167.586(91.895-416.417) | 3567.849(2263.415-5348.257) | 155.369(98.565-232.901) | -0.073(-0.624-0.795) | -0.21(-0.33,-0.09) |
| Lebanon | 206.295(134.405-316.369) | 19.725(12.851-30.249) | 154.506(103.536-257.683) | 12.089(8.101-20.162) | -0.387(-0.639--0.000) | -1.21(-1.56,-0.85) |
| Libya | 405.192(260.872-680.256) | 22.375(14.405-37.564) | 259.672(173.912-396.766) | 17.409(11.659-26.600) | -0.222(-0.542-0.301) | -0.58(-0.69,-0.46) |
| Malaysia | 5075.008(3397.597-8502.632) | 77.210(51.690-129.357) | 3838.994(2669.604-5951.970) | 50.423(35.063-78.175) | -0.347(-0.590-0.037) | -1.17(-1.34,-0.99) |
| Maldives | 94.276(50.686-161.841) | 89.751(48.254-154.074) | 34.635(24.183-47.195) | 34.577(24.142-47.115) | -0.615(-0.801--0.194) | -2.91(-3.10,-2.72) |
| Mauritius | 169.901(149.820-195.197) | 51.477(45.393-59.141) | 172.309(141.173-204.337) | 83.079(68.067-98.521) | 0.614(0.266-1.028) | 1.82(1.35,2.30) |
| Mongolia | 3498.691(1926.393-6026.669) | 388.741(214.042-669.625) | 1852.518(1104.540-3027.056) | 170.487(101.651-278.580) | -0.561(-0.786--0.075) | -2.07(-2.32,-1.82) |
| Morocco | 4100.617(2305.428-8356.077) | 373.190(94.792-631.435) | 2710.333(1547.502-4189.820) | 248.403(115.852-418.772) | -0.334(-0.644-0.760) | -1.65(-1.92,-1.37) |
| Myanmar | 27916.482(12940.894-49289.923) | 188.927(87.579-333.573) | 25716.559(12049.291-40990.297) | 164.694(77.166-262.510) | -0.128(-0.599-0.749) | -0.39(-0.60,-0.18) |
| Nepal | 43313.431(21240.076-72621.395) | 514.095(252.102-861.957) | 19734.519(9121.289-31304.027) | 213.873(98.852-339.257) | -0.584(-0.770--0.220) | -2.83(-2.96,-2.69) |
| Oman | 2134.844(1383.922-3207.680) | 254.035(164.679-381.697) | 589.347(383.427-1157.217) | 48.188(31.351-94.620) | -0.810(-0.906--0.589) | -5.50(-5.94,-5.06) |
| Pakistan | 164286.190(102814.566-220506.887) | 333.630(208.794-447.802) | 259490.401(135736.034-389917.661) | 303.703(158.863-456.353) | -0.090(-0.422-0.460) | 0.23(0.01,0.44) |
| Palestine | 181.997(126.472-277.373) | 18.081(13.116-33.100) | 498.395(329.395-702.468) | 17.966(12.961-23.937) | -0.006(-0.568-0.511) | -0.20(-0.84,0.44) |
| Philippines | 56403.274(38157.384-78761.387) | 223.695(151.332-312.367) | 62902.940(36595.655-81989.963) | 185.017(107.639-241.157) | -0.173(-0.475-0.103) | -0.37(-0.55,-0.18) |
| Qatar | 36.681(20.557-56.953) | 29.335(16.440-45.547) | 44.812(27.571-64.954) | 9.073(5.582-13.151) | -0.691(-0.820--0.481) | -3.70(-4.06,-3.34) |
| Republic of Korea | 2055.929(1491.312-3763.613) | 16.999(13.109-21.634) | 1091.352(787.342-1454.035) | 9.075(6.479-11.922) | -0.466(-0.616--0.253) | -1.15(-1.99,-0.29) |
| Saudi Arabia | 4981.150(3146.923-8512.779) | 76.009(48.020-129.899) | 1634.617(910.636-3335.037) | 21.607(12.037-44.084) | -0.716(-0.878--0.423) | -4.91(-5.39,-4.43) |
| Seychelles | 3.401(2.446-6.365) | 14.335(10.309-26.825) | 6.353(3.631-8.986) | 27.142(15.515-38.391) | 0.893(-0.258-2.135) | 2.02(0.92,3.14) |
| Singapore | 256.089(224.904-292.103) | 195.021(97.555-361.522) | 193.850(159.952-230.153) | 144.773(89.122-216.871) | -0.258(-0.630-0.527) | -1.15(-1.24,-1.05) |
| Socialist Republic of Viet Nam | 93316.095(54674.343-141171.854) | 351.971(206.221-532.474) | 55979.156(28519.386-88892.553) | 226.063(115.171-358.979) | -0.358(-0.663-0.279) | -1.01(-1.24,-0.77) |
| Sri Lanka | 5344.560(4007.975-9041.240) | 96.593(72.437-163.404) | 2496.387(1582.834-4618.218) | 48.910(31.012-90.482) | -0.494(-0.675--0.234) | -2.34(-2.72,-1.95) |
| Sudan | 7070.731(2743.935-13975.132) | 41.904(23.559-85.390) | 7919.363(4747.806-12678.681) | 27.679(15.804-42.789) | -0.339(-0.642-0.216) | -0.86(-1.14,-0.58) |
| Syrian Arab Republic | 4134.003(2790.840-6020.333) | 69.806(47.126-101.659) | 1904.288(1264.591-2647.829) | 51.983(34.520-72.280) | -0.255(-0.624-0.192) | -1.37(-1.80,-0.94) |
| Taiwan (Province of China) | 1966.483(1761.147-2198.114) | 174.518(97.570-247.748) | 834.494(708.276-975.084) | 53.591(38.660-87.636) | -0.693(-0.819--0.289) | -5.12(-5.76,-4.47) |
| Tajikistan | 181.532(95.583-315.145) | 7.818(4.116-13.572) | 237.724(133.192-379.947) | 6.632(3.716-10.600) | -0.152(-0.553-0.590) | -0.50(-0.71,-0.28) |
| Thailand | 29421.210(16448.936-41766.751) | 238.507(201.754-286.710) | 5233.840(3775.624-8558.824) | 145.988(116.171-183.703) | -0.388(-0.546--0.194) | -1.44(-1.80,-1.08) |
| Timor-Leste | 912.763(321.632-1643.037) | 274.408(96.693-493.952) | 1113.443(624.796-1696.309) | 213.860(120.005-325.812) | -0.221(-0.624-0.690) | -1.24(-1.45,-1.02) |
| Tunisia | 1088.985(736.380-1742.852) | 35.067(23.712-56.122) | 572.258(378.392-847.193) | 20.691(13.681-30.632) | -0.410(-0.653--0.005) | -1.13(-1.35,-0.91) |
| Turkey | 11847.931(7414.905-19152.155) | 57.827(36.190-93.477) | 4496.723(3203.548-6061.663) | 24.280(17.297-32.730) | -0.580(-0.752--0.306) | -2.64(-3.09,-2.20) |
| Turkmenistan | 1536.534(1170.730-1961.884) | 102.377(78.004-130.717) | 3781.306(2729.859-5109.156) | 248.117(179.124-335.246) | 1.424(0.600-2.740) | 3.43(2.32,4.54) |
| United Arab Emirates | 83.824(55.203-118.817) | 14.222(9.366-20.159) | 75.504(51.907-97.822) | 5.640(3.877-7.307) | -0.603(-0.744--0.389) | -2.13(-2.53,-1.73) |
| Uzbekistan | 26047.388(19137.337-35556.168) | 304.447(223.681-415.588) | 18536.041(13887.814-24730.557) | 183.688(137.625-245.074) | -0.397(-0.607--0.063) | -1.21(-1.59,-0.83) |
| Yemen | 5194.287(1609.789-9294.899) | 73.217(22.691-131.018) | 7934.183(3906.822-12893.758) | 57.542(28.334-93.511) | -0.214(-0.567-0.514) | -0.47(-0.80,-0.13) |
